# In Situ Photo‑Crosslinked Functional Bilayer Hydrogel Modulates Local Microenvironment and Remyelination After Optic Nerve Injury

**DOI:** 10.1002/advs.78031

**Published:** 2026-09-27

**Authors:** Tonghe Pan, Yate Huang, Junhao Zhang, Shudong Tian, Zhixing Li, Xuanwen Li, Yanchen Zhang, Dongmei Wang, Sihao Ye, Ming Jin, Neng Ling, Wentao Yan, Kaihui Nan, Wencan Wu

**Affiliations:** ^1^ Eye Research Center, Hangzhou Institute of Medicine, Chinese Academy of Sciences, Eye Hospital Wenzhou Medical University Hangzhou Zhejiang China; ^2^ Oujiang Laboratory (Zhejiang Lab for Regenerative Medicine, Vision and Brain Health) Wenzhou Zhejiang China; ^3^ State Key Laboratory of Eye Health, Eye Hospital Wenzhou Medical University Wenzhou Zhejiang China; ^4^ National Engineering Research Center of Ophthalmology and Optometry Institute of Biomedical Engineering, Eye Hospital Wenzhou Medical University Wenzhou Zhejiang China; ^5^ Zhejiang Key Laboratory of Key Technologies for Visual Pathway Reconstruction Wenzhou Medical University Wenzhou Zhejiang China

**Keywords:** bilayer hydrogel, clemastine, mesoporous polydopamine nanoparticles, microenvironment modulation, myelin preservation, optic nerve injury, RGC protection

## Abstract

Optic nerve injury often causes irreversible vision loss due to limited self‐regeneration capacity, persistent neuroinflammation, and failed remyelination. Current therapies partially promote axonal regeneration but rarely achieve meaningful visual recovery, limited mainly by unstable myelin reconstruction and inadequate injury microenvironment modulation. Here, we developed a photo‐crosslinkable bilayer hydrogel system (GO/GI@MPDA‐Cle). Its outer layer (GM‐Odex) provides strong wet tissue adhesion for optic nerve sheath sealing, while the inner layer (GM‐imid) enables sustained local release of drug‐loaded nanoparticles (MPDA‐Cle) for microenvironment modulation. In Vitro, the system exhibited excellent antioxidant and anti‐inflammatory properties and polarized microglia toward an anti‑inflammatory phenotype. In a rat partial optic nerve injury model, the hydrogel protected retinal ganglion cells through local immunomodulation and was associated with improved oligodendrocyte‐associated maturation and remyelination‐associated repair, accompanied by partial improvement in optic nerve conduction‐related function. Transcriptomic analysis further revealed that the treatment downregulated pathways related to inflammation, oxidative stress, and neuronal apoptosis, while upregulating genes involved in neuronal regeneration, axonal growth, and myelination. By integrating physical sealing, immunomodulation, and myelin repair, this study proposes a promising multifunctional strategy for optic nerve injury treatment.

## Graphical abstract

A blue‐light‐crosslinked bilayer hydrogel was developed for local optic nerve repair. The adhesive GM‐Odex outer layer seals and anchors the lesion, while the non‐adhesive GM‐imid inner layer releases MPDA‐Cle without obstructing CSF flow. This system modulates oxidative and inflammatory microenvironments, supports oligodendrocyte differentiation and remyelination, protects RGCs, and partially improves optic nerve conduction.

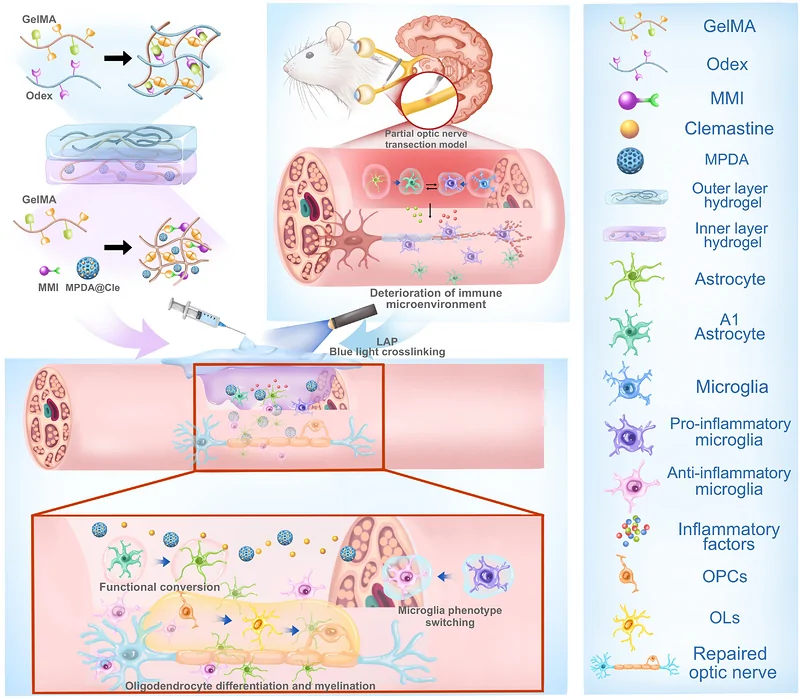

## 1 Introduction

Vision is the primary sensory interface through which humans perceive and interact with the world [1]. As a specialized extension of the central nervous system (CNS), the optic nerve serves as the indispensable “conduit” for transmitting visual information from the retina to the brain. However, the mammalian optic nerve possesses negligible intrinsic regenerative capacity [2, 3]. Injuries resulting from glaucoma or traumatic optic neuropathy (TON) trigger a devastating pathological cascade, characterized by persistent secondary damage, including retinal ganglion cell (RGC) apoptosis, progressive axonal degeneration, and chronic demyelination [4, 5]. Ultimately, these cumulative insults lead to the irreversible collapse of the visual system's structural and functional integrity [6].

In the past decade, significant strides have been made in stimulating optic nerve axonal regrowth via neurotrophic factor delivery, exosome therapy, or gene editing [7, 8]. Despite these morphological successes, the transition from axonal elongation to meaningful visual restoration remains a formidable therapeutic bottleneck. A primary obstacle is the failure of newly regenerated axons to undergo remyelination [5, 9]. Myelin sheaths are essential for high‐fidelity saltatory conduction; without them, nascent axons remain “electrically silent” and incapable of establishing functional neural circuits [10]. Successful remyelination hinges on the recruitment and maturation of oligodendrocyte precursor cells (OPCs) within a permissive local microenvironment [11, 12].

While current mainstream research focuses on intravitreal delivery to protect and regenerate retinal neurons, the local microenvironment at the primary lesion site in the optic nerve is often overlooked [8, 13, 14]. In reality, the lesion site is a hostile biochemical injury‐associated inflammatory microenvironment where injured axons release damage‐associated molecular patterns (DAMPs) and myelin breakdown products generate inhibitory fragments [15]. These factors collectively activate resident microglia toward a proinflammatory state unleashing neurotoxic mediators such as cytokines, reactive oxygen species, and nitric oxide that exacerbate neuronal death and directly stall myelin repair [16, 17]. Consequently, precisely regulating the local microenvironment at the injury epicenter is essential for preserving surviving axons and enabling functional remyelination [18].

Achieving localized therapy for the optic nerve is hindered by its deep anatomical location at the cranial base and the protection of the blood‐brain barrier. Systemic and intraocular administrations often fail to reach effective concentrations at the lesion site due to compromised axoplasmic transport and blood supply. While surgical decompression can alleviate mechanical pressure, it introduces a critical clinical dilemma between the imperative to hermetically seal the optic nerve sheath to prevent cerebrospinal fluid (CSF) leakage and the necessity of maintaining the patency of the subarachnoid space [19]. Standard tissue adhesives or monolithic hydrogels often fail this balance because excessive swelling or uncontrolled adhesion can obliterate the delicate subarachnoid gap. This obstruction of CSF microcirculation can lead to localized intracranial hypertension or secondary compressive ischemia [20, 21, 22].

Inspired by the “bandage” concept, we proposed a bilayer hydrogel system designed to manage this complex fluid‐structure interface. This design features an outer layer with robust tissue adhesiveness to reconstruct the integrity of the sheath and an inner layer that maintains a non‐adhesive soft interface with the nerve functioning as a physiological spacer that preserves the CSF flow pathway while serving as a bio‐active reservoir for sustained drug delivery without mechanical irritation.

To realize this multifunctional platform, we utilized Gelatin methacrylate (GelMA) as the primary scaffold. To overcome the poor wet‐tissue adhesion of pure GelMA under the continuous pulsatile pressure of CSF, oxidized dextran (ODex) was integrated to form a dual‐network structure (GM‐Odex) significantly enhancing mechanical robustness and interfacial bonding via Schiff base reactions [23, 24, 25].

For the inner layer, GelMA was crosslinked with 2‐mercapto‐1‐methylimidazole (MMI) via a thiol‐ene click reaction (GM‐imid). In the present system, the introduction of 2‐mercapto‐1‐methylimidazole was mainly intended to regulate the network structure, crosslinking degree, and mechanical properties of the inner hydrogel, thereby constructing an inner layer better suited as a non‐adhesive local therapeutic interface. This design not only tailored the hydrogel stiffness to match neural tissue but may also provide a modest anti‐inflammatory contribution from the imidazole‐containing component [26]. To actively remodel the pathological microenvironment, mesoporous polydopamine (MPDA) nanoparticles were incorporated owing to their superior radical scavenging and immune‐modulating capabilities [3, 27, 28]. Finally, clemastine, a potent M1 muscarinic receptor antagonist that promotes OPC differentiation, was loaded into the MPDA mesopores. This configuration (GO/GI@MPDA‐Cle) enables the coordinated release of therapeutic agents directly at the injury epicenter [29, 30, 31].

Critically, the structural hierarchy of this bilayer system is finalized through rapid in situ blue‐light crosslinking which allows for precise spatial conformality to the curvature of the sheath without encroaching upon the nerve itself. The explicitly engineered non‐adhesive nature of the inner layer ensures that the hydrogel does not fuse the nerve to the sheath. This maintains the natural hydrodynamic environment of the subarachnoid space and eliminates the risk of secondary intracranial hypertension.

**SCHEME 1:**
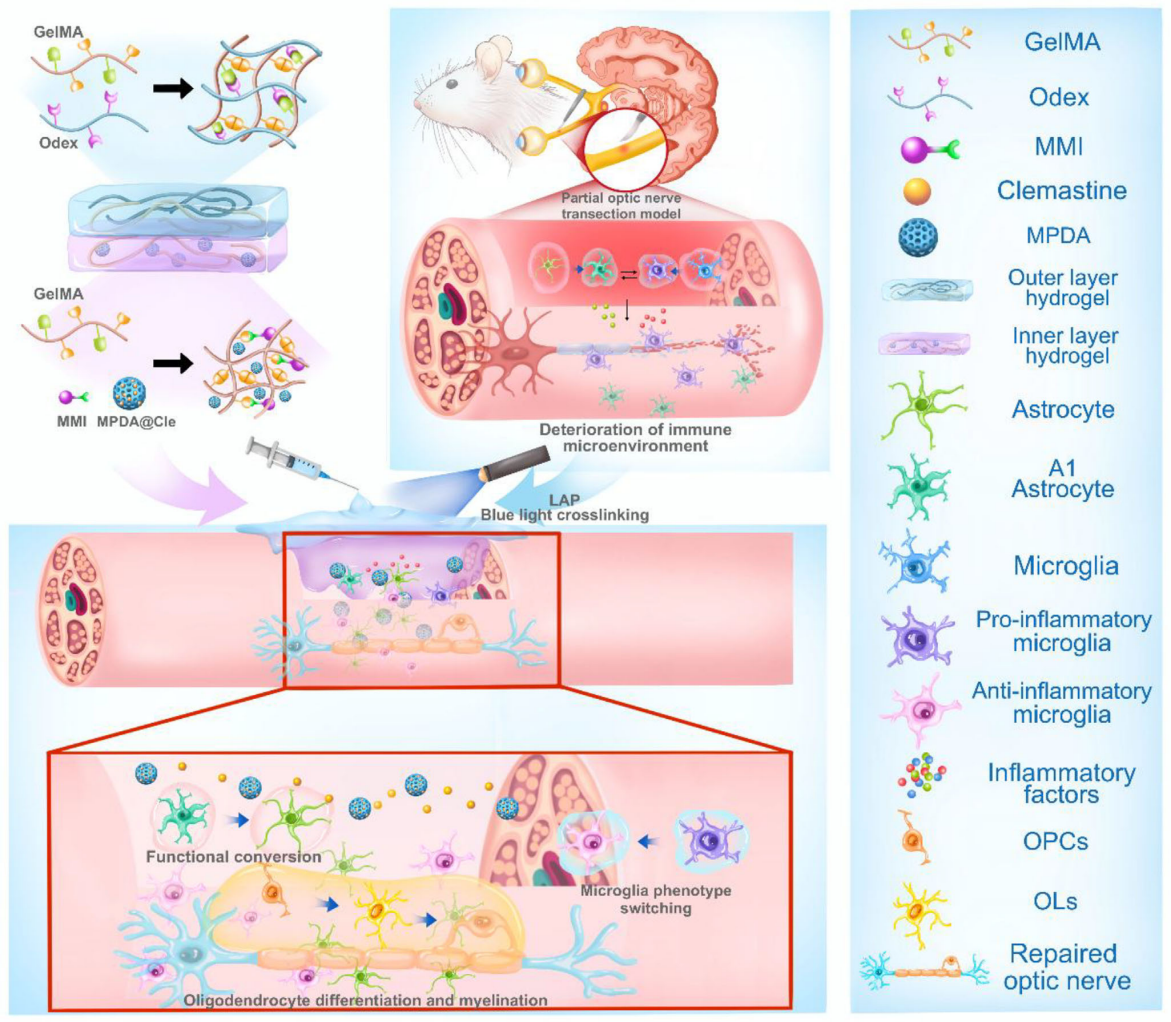
Schematic illustration of the synthesis of the GM‐Odex/GM‐imid@MPDA‐Cle (GO/GI@MPDA‐Cle) bilayer hydrogel and its dual‐functional application for sealing the optic nerve sheath and modulating the local microenvironment in partial optic nerve injury.

In this study, we demonstrate that the GO/GI@MPDA‐Cle bilayer system provides a sophisticated solution for optic nerve repair by simultaneously addressing biological regeneration and physiological fluid homeostasis. By integrating a physical barrier, sustained immunomodulation, and pro‐myelinating cues, this “all‐in‐one” platform effectively preserves surviving axons and promotes functional recovery. We anticipate that this bilayer hydrogel design offers a sophisticated and translationally relevant strategy for the comprehensive management of traumatic optic nerve injuries while establishing novel strategies for other CNS repair scenarios where the maintenance of CSF dynamics is of paramount importance. (Scheme 1).

## 2 Materials and Methods

### 2.1 Materials

GelMA (60% degree of substitution, DS60) was procured from Shuhe Biotechnolo‐gy Co. (China). 2‐Mercapto‐1‐methylimidazole (2‐MMI), diethylene glycol, sodium periodate (NaIO4), lithium phenyl‐2,4,6‐trimethylbenzoylphosphinate (LAP), and sucrose were purchased from Macklin (China). Dextran, dopamine hydrochloride, Pluronic F127, interferon‐gamma (IFN‐γ) protein, and lipopolysaccharide (LPS) were supplied by Sigma–Aldrich (USA). 2',7'‐Dichlorodihydrofluorescein diacetate (DCFH‐DA) and Cholera Toxin Subunit B (CTB) were obtained from Thermo Fisher Scientific (USA). The Cell Counting Kit‐8 (CCK‐8) and Calcein‐AM/PI double stain kit were acquired from DOJINDO Laboratories (Japan). Dulbecco's Modified Eagle Medium/Nutrient Mixture F‐12 (DMEM/F‐12), low glucose DMEM, trypsin, and fetal bovine serum (FBS) were purchased from Gibco (USA). All other chemicals were used without further purification.

### 2.2 Synthesis of ODex and Preparation of Hydrogels

ODex was synthesized as previously reported [32]. Briefly, dextran (2 g) was dissolved in deionized water to obtain a 10% (w/v) solution. NaIO4 (1.6 g) was then added, and the reaction was stirred at room temperature for 3 h protected from light. An equimolar amount of diethylene glycol was added after oxidation. The reacted solution was dialyzed against deionized water for 3 days, and ODex powder was obtained by lyophilization. The oxidation degree (OD) of ODex was measured as described [24]. For the assay, a known amount of ODex was dis‐solved in 25 mL of 0.25 mol/L hydroxylamine hydrochloride containing 0.05% methyl orange and left at room temperature for 3 h. The mixture was titrated with standard NaOH to the red‑to‑yellow end point. OD was calculated as the following equation:

(1) $$\mathrm{OD}=\frac{\left(V_{1}-V_{0}\right)\times M\times M_{W}}{w}\times 100$$

where *V_0_* is the NaOH volume for the blank, *V_1_* is the NaOH volume for the sample, *M* is the NaOH molarity, *M_W_* is the molecular weight of a dextran glucose unit, and *W* is the weight (g) of ODex. Results are the average of five batches.

For hydrogel preparation, LAP photoinitiator was first dissolved in phosphate‐buffered saline (PBS) to 0.25% (w/v) and heated at 55 °C. GelMA was then dissolved in this LAP solution to achieve final GelMA concentrations of 10%, 15%, or 20% (w/v). ODex was added to produce pre‐hydrogels with the following GelMA:ODex mass ratios of 10:0 (G10), 10:2 (G10OD2), 10:5 (G10OD5), 15:0 (G15), 15:2 (G15OD2), 15:5 (G15OD5), 20:0 (G20), 20:2 (G20OD2), and 20:5 (G20OD5). The pre‐hydrogel mixtures rapidly formed Schiff bases upon mixing and were then photo crosslinked with visible light (405 nm, 30 mW/cm^2^) for 1 min using a blue light source.

GM‐imid hydrogels were synthesized by grafting 2‐mercapto‐1‐methylimidazole on‐to GelMA via click chemistry, followed by blue‐light crosslinking. In this process, GelMA was dissolved in PBS to form a solution, which was then supplemented with 2‐mercapto‐1‐methylimidazole and the photo‐initiator LAP at 0.25% (w/v). The resulting prepolymer mixtures, with GelMA‐to‐imidazole mass ratios of 10:2 (G10imid2), 10:5 (G10imid5), 15:2 (G15imid2), 15:5 (G15imid5), 20:2 (G20imid2), and 20:5 (G20imid5), were irradiated with blue light for 1 min to form the final GM‐imid hy‐drogels.

### 2.3 Synthesis of MPDA NPs and MPDA‐Cle NPs

MPDA NPs were synthesized via a template method [28]. In brief, Pluronic F127 (0.5 g), dopamine hydrochloride (0.5 g), and 1,3,5‐trimethylbenzene (TMB, 0.8 mL) were ultrasonically dispersed in a mixture of DI water (25 mL) and ethanol (25 mL) to form an emulsion. Ammonia (2 mL) was added to the reaction mixture while stirring at 40 °C. After 3 h, the MPDA NPs were collected by centrifugation at 12,000 rpm and washed several times with a mixture of water, ethanol, and acetone. The purified NPs were obtained by lyophilization.

For drug loading, MPDA NPs were dispersed in DI water (10 mg/mL). This solution (1 mL) was mixed with clemastine fumarate solution (2 mg/mL, 1 mL) and stirred in the dark for 12 h to enable drug loading through electrostatic adsorption and hydrophobic interactions. The loaded particles, designated MPDA‐Cle, were collected by centrifugation (12,000 rpm, 5 min), washed three times with DI water to remove unbound drug, and redispersed in DI water. Drug loading efficiency was determined by measuring the supernatant's absorbance using UV‐Vis‐NIR spectroscopy and calculated using the following equations:

(2) L=O−R

(3) $$Drug\;Loading\;Content\left(\%\right)=\frac{L}{L+M}\times 100$$

where *L* is the loading clemastine fumarate content, *O* is the original Clemastine fumarate content, *R* is the residual clemastine fumarate content, *M* is the dispersed MPDA NPs content.

### 2.4 Characterization

The hydrodynamic size distribution of MPDA NPs and MPDA‐Cle was measured by dynamic light scattering (DLS, Malvern Instruments, UK) at 25 °C. The morphologies of MPDA NPs and MPDA‐Cle were analyzed by transmission electron microscopy (TEM, JEOL, Japan) at 200 kV. Nitrogen adsorption/desorption isotherms were measured with an ASAP2460 analyzer (Micromeritics, USA). Hydrogel morphologies were characterized using scanning electron microscopy (SEM, Thermo Fisher Scientific, USA). UV‐Vis‐NIR absorption spectra were acquired using a UV‐Vis‐NIR spectrophotometer (UV‐1780, Shimadzu, Japan). Chemical structures were verified by Fourier transform infrared spectroscopy (FTIR, Nicolet iS50, Thermo Fisher Scientific, USA). Rheological properties were measured using a DHR‐2 rheometer (TA Instruments, USA). Cell viability was quantified using a SpectraMax M5 microplate reader (Molecular Devices, USA). Fluorescence images were acquired using a confocal microscope (Stellaris5, Leica, Germany).

### 2.5 Swelling Test

The swelling ratio of the hydrogels was determined by gravimetric analysis. Briefly, the hydrogels (initial mass recorded as *W_0_*) were immersed in PBS at 37 °C. At predetermined time intervals, the samples were removed, the surface moisture was gently blotted with filter paper, and the wet mass was immediately measured (*W_1_*). The swelling ratio (*SR*) was then calculated using the following equation:

(4) $$SR=\frac{\left(W_{1}-W_{0}\right)}{W_{0}}\times 100\%$$

### 2.6 Rheological Studies

The rheological properties of hydrogels were characterized using a DHR‐2 rheometer (TA Instruments, USA) with 25 mm parallel plates and a Peltier temperature‐control system at 25 °C. Frequency sweep measurements were conducted from 0.1 to 100 rad/s at a fixed strain of 1% to assess the mechanical behavior. Furthermore, time sweep tests were performed at a frequency of 1 Hz and 1% strain to evaluate the hydrogel stiffness under identical temperature conditions.

### 2.7 In Vitro Degradation of the Hydrogels

The In Vitro degradation profiles of the hydrogels were evaluated by monitoring mass loss in PBS. Cylindrical samples were incubated in PBS containing collagenase (1 µg/mL) and weighed at predetermined intervals. To further mimic the slow cerebrospinal fluid (CSF) motion in the optic nerve subarachnoid space, a microinjection system was also used to provide continuous fluid perfusion at a low flow condition corresponding to the reported human ONSAS CSF velocity range (approximately 0.5‐0.8 mm/s) [33]. The PBS solution was refreshed after each weighing, and the samples were returned for continued incubation until complete degradation occurred. The degradation rate (*DR*) was calculated using the formula:

(5) $$DR=\frac{\left(W_{0}-W_{t}\right)}{W_{0}}\times 100\%$$

where *W_0_* is the initial weight of the hydrogels and *W_t_* is the weight at the corresponding time point. All experiments were performed in triplicate.

### 2.8 Evaluation of Mechanical Properties

Rectangular hydrogel specimens (30 mm in length, 10 mm in width, 1 mm thick) were prepared for tensile testing. Prior to testing, the samples were equilibrated in PBS (pH 7.4) for 1 h to reach hydration equilibrium. After blotting the surface moisture with filter paper, each end of a sample was bonded to a glass slide using cyanoacrylate adhesive. The glass slides were then mounted in the grips of a texture analyzer (CTX, Brookfield Ametek, USA). Uniaxial tensile tests were performed at a crosshead speed of 6 mm/min with an initial gauge length of 10 mm. The tensile strength, elastic modulus, and elongation at break were determined directly from the stress‐strain curves without prior preconditioning. Each reported value represents the mean of at least five independent measurements.

### 2.9 In Vitro Adhesion Tests

In Vitro burst pressure tests were performed on porcine optic nerve dura mater. Square dura specimens (1.5 × 1.5 cm) were dissected from fresh porcine optic nerves. A 4 mm full‐thickness incision was created in the center of each specimen using a 15° ophthalmic knife (Alcon, USA). The specimen was then mounted on custom‐built burst pressure device. A 15 µL aliquot of the pre‐hydrogels was applied to the incision site and crosslinked in situ for 1 min. Saline was continuously infused into the device at a rate of 5 mL/h using a syringe pump (TJ‐3A, Lange Precision Pump Co., China). The burst pressure was recorded with a manometer (Dongguan Xintai Instrument Co., China) and defined as the maximum pressure achieved before fluid leakage occurred from the wound.

### 2.10 ABTS Assay

ABTS^•+^ radical scavenging activity was measured following the manufacturer's protocol with minor modifications. The ABTS^•+^ working solution containing ABTS^•+^ and oxidant was reacted overnight at room temperature in the dark. The resulting ABTS^•+^ solution was then adjusted with PBS to an absorbance of 0.7 ± 0.05 at 734 nm. Next, 100 µL of each sample was mixed with 900 µL of the ABTS^•+^ The seven sample groups were: (1) phosphate‐buffered saline (PBS); (2) GM‐Odex; (3) GM‐imid; (4) GM‐imid@MPDA (1 mg/mL, *based on MPDA*); (5) GM‐imid@MPDA‐Cle (1 mg/mL, *based on MPDA*); (6) MPDA (1 mg/mL); and (7) Clemastine (150 µg/mL). The color change was documented with a phone camera over 3 h. After this period, the absorbance at 734 nm was recorded using a SpectraMax M5 microplate reader (Molecular Devices, USA)

### 2.11 In Vitro Release of Clemastine

The In Vitro release profile of clemastine from GM‐imid@MPDA‐Cle hydrogel (G20imid5, containing 1 mg/mL MPDA‐Cle) was investigated. Briefly, 2 mL of the blue‐light‐crosslinked GM‐imid@MPDA‐Cle hydrogel was placed into a dialysis bag, which was then immersed in a centrifuge tube containing 10 mL of PBS. A control group with an equivalent amount of free clemastine was established based on the drug loading within the GM‐imid@MPDA‐Cle hydrogel. The tubes were incubated in a shaking incubator at 37 °C and 100 rpm. At predetermined time points, 2 mL of the release medium was collected and replaced with an equal volume of fresh PBS. The concentration of clemastine in the collected samples was analyzed using a UV‐Vis‐NIR spectrophotometer (UV‐1780, Shimadzu, Japan). All samples were filtered through a 0.22 µm syringe filter (Membrane Solutions, USA) prior to analysis.

### 2.12 Cell Cultures

The retinal precursor cell line R28, provided by the Department of Anatomy and Neurobiology (Central South University, China), was used for In Vitro studies of retinal ganglion cell (RGC) neuroprotection and function [34]. The oligodendrocyte precursor cell line OLN‐93, derived from rats, was obtained from the Key Laboratory of Eye & Brain Connection (Wenzhou Medical University, China). The murine microglial cell line BV2 (CL‐0493) was sourced from Procell Life Science & Technology Co., Ltd. (Wuhan, China). All cell lines were cultured in DMEM or DMEM/F12 medium supplemented with 10% fetal bovine serum (FBS) and 1% penicillin‐streptomycin at 37 °C in a 5% CO_2_ atmosphere.

### 2.13 In Vitro Cytotoxicity

The cytotoxicity of hydrogels was evaluated using CCK‐8 and Live/Dead stain assays. Each prehydrogel solution (GM‑Odex, GM‑imid, and GO/GI@MPDA‑Cle) was added to separate wells of a 96‑well plate and crosslinked under blue light (405 nm) for 1 min. After gelation, the hydrogels were washed three times with PBS. R28 cells in the exponential growth phase were then seeded onto the hydrogels at a density of 1 × 10^4^ cells per well in 100 µL of medium and cultured at 37 °C under 5% CO_2_. After 24, 48, and 72 h of culture, the medium was replaced with 100 µL of fresh medium containing 10% CCK‐8 reagent. The plates were further incubated for 1 h. The absorbance at 450 nm was measured using a microplate reader. Cell viability was calculated as follows:

(6) $$Cell\;Viability\left(\%\right)=\frac{\left(A_{e}-A_{blank}\right)}{\left(A_{c}-A_{blank}\right)}\times 100\%$$

where *A_e_* and *A_c_* represent the absorbance values of the experimental and the control specimens at 450 nm, respectively.

For the Live/Dead stain assay, R28 cells were seeded in a 24‐well plate and cultured for 24 h. After blue‑light crosslinking, each hydrogel (GM‑Odex, GM‑imid, and GO/GI@MPDA‑Cle) was co‑cultured separately with cells in Transwell wells for 24, 48, or 72 h. The cells were stained with Calcein‑AM/PI for 30 min and imaged with a fluorescence microscope (DMi8, Leica, Germany).

### 2.14 Intracellular ROS Detection

R28 cells were seeded on coverslips in a 24‐well plate at a density of 1 × 10^4^ cells per coverslip for 24 h. After the culture medium was removed, the cells were incubated with DCFH‐DA for 6 h at 37 °C in the dark. The cells were then washed with PBS to remove excess DCFH‐DA and were subsequently incubated with DMEM containing 10% FBS and 100 µM H_2_O_2_. Meanwhile, GO/GI, GO/GI@MPDA, or GO/GI@MPDA‑Cle hydrogels were placed in Transwell inserts and added to the 24‐well plate, followed by co‐incubation for 2 h at 37 °C. Cells without any treatment and those treated with only H_2_O_2_ were used as the negative and positive controls, respectively. Fluorescence images were collected using a confocal microscope (Stellaris5, Leica, Germany).

### 2.15 In Vitro Microglia Phenotype Regulation

BV2 cells were seeded on coverslips in a 24‐well plate at 3 × 10^4^ cells per coverslip and cultured for 24 h. After 24 h, the medium was replaced with DMEM containing LPS (100 ng/mL) and IFN‐γ (20 ng/mL) to induce pro‐inflammatory polarization. Subsequently, Transwell inserts containing GO/GI, GO/GI@MPDA, or GO/GI@MPDA‐Cle hydrogels (n = 5) were placed in the same 24‐well plates, and co‐incubation was continued for another 24 h at 37°C. Cells without any treatment served as the control group. The expression of pro‐inflammatory (CD86) and anti‐inflammatory (CD206) surface markers in BV2 cells, as well as inflammatory cytokines (TNF‐α and IL‐6), was examined by immunofluorescence, and fluorescence images were acquired using a confocal microscope (Stellaris5, Leica, Germany).

### 2.16 In Vitro Inflammatory Signaling Analysis in LPS‐Induced Microglia

To further evaluate inflammation‐related signaling, BV2 microglia were stimulated with LPS and treated using a Transwell‐based system. The experimental groups included Control, Model, GO/GI, MPDA, GO/GI@MPDA, and GO/GI@MPDA‐Cle. After treatment, cells were fixed with 4% paraformaldehyde, permeabilized, blocked, and incubated with primary antibodies against P65 (1:500,Selleck, G23D18) or IκBα (1:500, Proteintech, 66418‐1‐Ig), followed by fluorescent secondary antibodies. Nuclei were counterstained with DAPI, and images were acquired using a fluorescence microscope. Fluorescence intensity was analyzed using ImageJ.

For Western blot analysis, total protein was extracted from microglia after different treatments. Equal amounts of protein were separated by SDS‐PAGE and transferred onto PVDF membranes. After blocking, membranes were incubated with primary antibodies against NLRP3 (1:1000, Proteintech, 30109‐1‐AP), IL‐1β (1:1000, Proteintech, 66737‐1‐Ig), caspase‐1(1:1000, Proteintech, 22915‐1‐AP), and GAPDH (1:1000, Proteintech, 60004‐1‐Ig), followed by HRP‐conjugated secondary antibodies. Protein bands were visualized using an enhanced chemiluminescence system and quantified using ImageJ.

### 2.17 DNA Damage Detection

BV2 cells were seeded into Transwell inserts at 1 × 10^4^ cells per well and cultured for 24 h. After discarding the medium, the cells were incubated in DMEM supplemented with LPS (100 ng/mL) and IFN‐γ (20 ng/mL) to induce pro‐inflammatory polarization. Meanwhile, OLN‐93 cells were seeded on coverslips in a 24‐well plate at 3 × 10^4^ cells per coverslip and cultured for 24 h. The Transwell inserts containing pro‐inflammatory BV2 cells were then co‐incubated for 24 h with GO/GI, GO/GI@MPDA, or GO/GI@MPDA‐Cle hydrogels. After 24 h, Transwell inserts were transferred into the 24‐well plate containing OLN‐93 cells, and the group co‐incubated with BV2 cells that were not treated with LPS and IFN‐γ was considered the negative control. After 24 h of culture at 37°C, the cell coverslips were washed with PBS and stained with γ‐H2AX and DAPI (Beyotime, C2036S). Fluorescence images were acquired using a confocal microscope (Stellaris5, Leica, Germany).

### 2.18 In Vitro Blood Compatibility

Fresh rat red blood cells (RBCs) were isolated from healthy donors and diluted to 0.8 mL aliquots. Each aliquot was mixed with 0.2 mL of test solutions: 0.1% Triton X‐100 (positive control), PBS (negative control), uncrosslinked GO/GI, and uncrosslinked GO/GI@MPDA‐Cle. Following 1 h incubation at 37 °C, samples were centrifuged (8000 rpm, 5 min), and the supernatant absorbance at 540 nm was measured. The hemolysis ratio was calculated using the following formula:

(7) $$\mathrm{Hemolysis}\;\mathrm{ratio}\left(\%\right)=\frac{\left(A_{e}-A_{p}\right)}{\left(A_{t}-A_{p}\right)}\times 100\%$$

where *A_e_*, *A_p_*, and *A_t_* are the supernatant fraction absorbance of the experimental groups, PBS, and Triton X‐100, respectively.

### 2.19 Partial Optic Nerve Injury (PONI) Model and Surgical Procedures

Adult Sprague Dawley (SD) rats weighing 220–240 g were supplied by Jiesijie Experimental Animal Co., Ltd (China). The animals were housed under a 12‐h light/dark cycle with free access to food and water. For surgical procedures, anesthesia was induced with inhaled isoflurane. Euthanasia was performed by an intraperitoneal injection of 2.0% pentobarbital sodium. All animal experiments were approved by the Animal Protection and Use Committee of the Eye Hospital of Wenzhou Medical University (Approval No. YSG23121401) and complied with relevant guidelines.

PONI was induced according to previously reported protocols [35]. In brief, the optic nerve was exposed through a conjunctival incision based on the temporal lobe and fornix, and a blunt dissection was performed. A 31G needle tip was then used to carefully incise the optic nerve sheath longitudinally in the avascular dura mater region about 2 mm behind the eyeball, followed by an arachnoid tear. The optic nerve was partially transected by guiding the needle tip along a specialized device. In the control group, the optic nerve was exposed without any transection. Afterward, 10 µL of 37 °C‐preheated GO/GI hydrogel, GO/GI@MPDA hydrogel, or GO/GI@MPDA‐Cle hydrogel was applied to the partially transected area using a microsyringe, and then photo crosslinked with visible light (405 nm, 30 mW/cm^2^). An equivalent volume of clemastine solution was locally injected as the clemastine group to compare sustained drug delivery from the dual‐layer hydrogel. To confirm partial axonal transection, cholera toxin subunit B (CTB), a classic anterograde tracer, was injected into the vitreous chamber 2 days before euthanasia (1 µg/µL, 10 mM PBS, 5 µL).

### 2.20 Immunofluorescence Labeling

After intracardiac perfusion with 4% PFA, the eyes and optic nerves were excised, post‐fixed in 4% PFA for 2 h, rinsed in 10 mM PBS for 5 min, and then immersed in 10% sucrose overnight. The tissues were embedded in OCT (Raymond A Lamb Ltd.) and frozen at –80 °C overnight. Consecutive 15 µm sections of the optic nerve and 8 µm sections of the eye cup were cut on a cryostat (CM 1950, Leica, Germany) and mounted on glass slides. Sections or cell coverslips were washed three times in 10 mM PBS, blocked in PBS containing 0.3% Triton X‐100 and 10% normal goat serum at room temperature for 1 h, and then incubated overnight at 4 °C with one or more of the following primary antibodies: rabbit polyclonal anti‐TNF‐α (1:500, Bioss, bs10802R), mouse monoclonal anti‐IL‐6 (1:500, Proteintech, 66146‐1‐Ig), rabbit polyclonal anti‐BRN3A (1:500, Bioss, bs‐3669R), mouse monoclonal anti‐CD206 (1:500, abcam, ab64693), mouse monoclonal anti‐CD86 (1:500, abcam, ab220188), rabbit monoclonal anti‐CD68 (1:500, abcam, ab283654), rat monoclonal anti‐MBP (1:500, Sigma–Aldrich, MAB386), mouse monoclonal anti‐APC (1:500, Sigma–Aldrich, OP80), and rabbit polyclonal anti‐NG2/CSPG4 (1:500, Sigma–Aldrich, AB5320). Slides or cell coverslips were washed three times in PBS and then incubated with Cy3, Alexa Fluor647 or Alexa Fluor488 (1:1000, Jackson Immuno Research) for 2 h at room temperature. After rinsing with PBS, slides were mounted in antifade medium containing DAPI (Beyotime). Fluorescence images were acquired on a confocal microscope (Stellaris5, Leica) or a fluorescence microscope (DM4B, Leica).

### 2.21 Quantification of RGCs

After euthanasia, retinas were collected and subjected to immunolabeling for RGC counting (n = 5). Flat‐mounted retinas were divided into four quadrants and examined under a fluorescence microscope (Cell Observer SD, Zeiss, Germany). In each quadrant, three areas located 2, 3, and 4 mm away from the optic disc were photographed at ×200 magnification. Brn3a‐labeled RGCs were counted in the images using ImageJ, and the density of labeled RGCs (cells/mm^2^) was determined by averaging twelve counts per retina.

### 2.22 Transcriptomic Profiling and Functional Enrichment Analysis

Differential gene expression analysis was performed in R using DESeq2 (v1.44.0) with raw count data as input. Genes meeting the criteria of |log2FC| > 1 and p‐value < 0.05 were designated as significantly differentially expressed (DEGs). Functional enrichment analysis of these DEGs was conducted using clusterProfiler (v4.12.2) to identify overrepresented GO biological processes and KEGG pathways. Terms and pathways with a p‐value < 0.05 were retained, with priority given to immune‐related categories. Data visualization included heatmaps and volcano plots for differential expression, as well as dot plots (bubble charts) and bar plots for enrichment results, all generated with ggplot2 (v3.5.2).

### 2.23 Statistical Analysis

All experiments were performed in quintuplicate unless otherwise indicated. Data are expressed as mean ± standard deviation (S.D.). One‐way ANOVA with Tukey's post hoc test (multiple group comparison) or Student's t test (two group comparison) was used to assess statistical significance at the 95% confidence level in GraphPad Prism 8.0. Significance thresholds were set at *p < 0.05, **p < 0.01, ***p < 0.001, and ns, no significance.

## 3 Results and Conclusions

### 3.1 Fabrication of Hydrogels

GelMA is widely used in tissue repair and sealing owing to its good biocompatibility and tunable physical properties [36, 37]. However, pure GelMA has limited functional groups for strong tissue bonding, and its single‐network structure often lacks sufficient stability in wet or pressurized environments, such as around the optic nerve. To overcome these limitations, interpenetrating polymer network (IPN) hydrogels have been developed [38, 39]. These materials typically combine two crosslinked networks, which improves mechanical strength, tissue adhesion, and stability under dynamic physiological conditions. For example, dual‐network hydrogels have shown effective sealing in moist cardiac tissues and compatible mechanical properties in vascular repair [40, 41].

**FIGURE 1:**
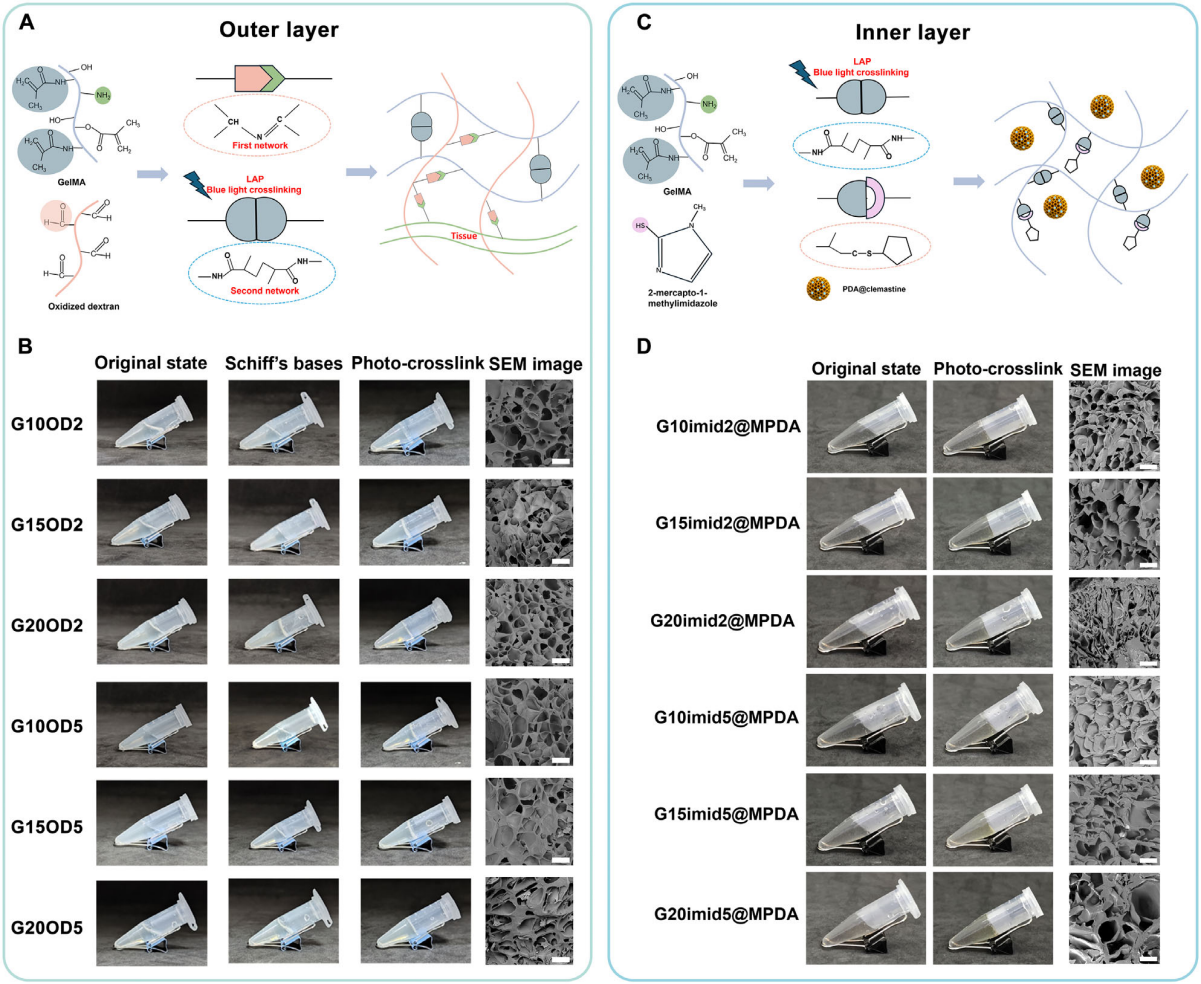
Fabrication and observation of dual‐layer hydrogels: outer layers with varying GelMA‐to‐ODex ratios and inner layers with different GelMA‐to‐2‐MMI ratios. (A) Schematic of synthesis of outer GM‐Odex adhesive hydrogel. (B) Overall comparison of hydrogel formation and SEM images at different GelMA:ODex ratios. (scale bars: 200 µm). (C) Schematic of synthesis of inner GM‐imid hydrogel. (D) Macroscopic appearance of hydrogel formation and representative SEM images at different GelMA:2‐MMI ratios. (scale bars: 200 µm).

In this study, a high‐performance, light‐triggered double‐network hydrogel was constructed by combining GelMA and ODex, leveraging the synergistic effects of both components (Figure 1A). GelMA provides a photopolymerizable backbone and biocompatibility, while the aldehyde groups of ODex can form covalent bonds with amino groups on tissue surfaces and reversibly react with amines on the GelMA chains via dynamic Schiff base linkages. This dynamic crosslinking mechanism endows the hydrogel with excellent tissue adhesiveness. More importantly, the second interpenetrating network, formed through blue‐light‐induced crosslinking, greatly enhances the adhesive properties and mechanical strength, enabling the hydrogel to withstand cerebrospinal fluid pressure and environmental stress around the optic nerve. When ODex (degree of oxidation: 105.89 ± 15.88%) is mixed with GelMA (GM‐Odex) in PBS at different ratios, the pre‐gel solution becomes viscous within 1 min at 37 °C due to initial Schiff base formation (Figure 1B), whereas GelMA without ODex remains fluid. This first Schiff base network confers preliminary gel‐like properties to the outer pre‐hydrogel, effectively resisting dilution in a wet environment and achieving rapid initial sealing to prevent cerebrospinal fluid leakage. Subsequent blue‐light irradiation initiates GelMA photocrosslinking, resulting in a stable double‐network hydrogel. A higher GelMA concentration further accelerates the gelation process. Scanning electron microscopy (SEM) shows that with higher GelMA concentrations and the introduction of ODex, the pore size of the hydrogel decreases to 20–200 µm, forming a denser three‐dimensional porous structure (Figure 1B).

To create an inner layer with drug‐controlled release, 2‐mercapto‐1‐methylimidazole was grafted onto GelMA chains (GM‐imid) via photo‐initiated thiol‐ene click chemistry (Figure 1C). This design enables regulation of the adhesive and mechanical properties of the hydrogel, while the imidazole‐containing network may provide a modest auxiliary contribution to local tissue protection [26]. The inner hydrogel contains mesoporous polydopamine nanoparticles (MPDA) loaded with the desired therapeutic agent, forming a localized sustained‐release system. The GM‐imid pre‐gel remains in a liquid state at 37 °C (Figure 1D), and solidifies rapidly within 1 min of blue‐light irradiation. The gelation rate is positively correlated with the GelMA concentration. Notably, due to the anatomical shielding of the optic nerve sheath, any unirradiated pre‐gel is naturally cleared away by cerebrospinal fluid, effectively avoiding intracranial hypertension associated with ectopic gel formation. Meanwhile, the solidified portion is firmly anchored by the outer hydrogel at the surgical window, thus enabling long‐term local drug delivery. SEM analysis indicates that GM‐imid exhibits a three‐dimensional porous architecture similar to GM‐ODex, which is favorable for sustained therapeutic release.

### 3.2 Characterization of Hydrogels

**FIGURE 2:**
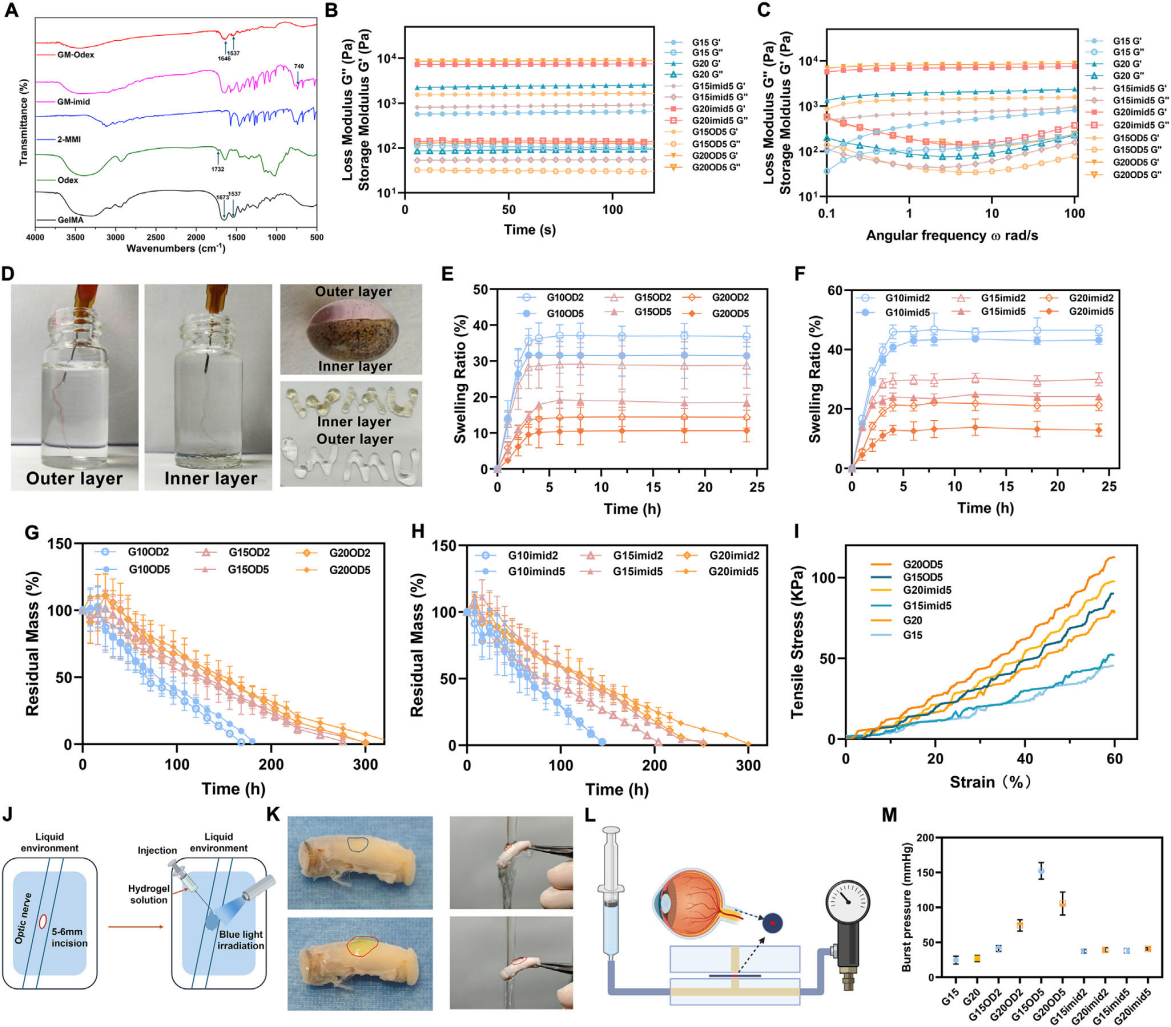
Chemical construction, rheological and mechanical characterization of the hydrogels. (A) FTIR spectra of GelMA, ODex, 2‐MMI, GelMA‐imid and GelMA‐Odex. (B) Time‐sweep and (C) Frequency‐sweep sequence of hydrogels. (D) The outer and inner hydrogels being extruded through a 27G needle in an aqueous environment (PBS) at 25 °C. (For ease of observation, a red dye was added to GM‐Odex.) Appearance of the outer and inner hydrogels adhering to each other. “WMU” written with the outer and inner hydrogels using a 27G needle. (E) Swelling ratio of GM‐Odex hydrogels and (F) GM‐imid hydrogels over time in PBS at 37 °C. (G) In Vitro degradation behavior of GM‐Odex hydrogels and (H) GM‐imid hydrogels in the presence of collagenase (1 µg/mL) under a mi‐croinjection system at 37 °C. (I) Representative tensile stress–strain curves of hydrogels. (J) Schematic illustration of GM‐Odex hydrogel application in a moist environment. (K) Macroscopic views of GM‐Odex hydrogel on fenestrated pig optic nerve sheaths under PBS conditions; the hydrogel remains adhered to the incision site after rinsing. (L) Schematic of the In Vitro setup for measuring hydrogel burst pressure using porcine optic nerve dura mater. (M) Burst pressure of hydrogels (n = 3).

Fourier transform infrared (FTIR) spectroscopy was employed to characterize the chemical structures of the hydrogels (Figure 2A). In the GM‐Odex composite, the characteristic carbonyl peak of ODex at 1732 cm^−1^ was significantly attenuated, while the primary amine absorption band of GelMA at 1537 cm^−1^ also showed markedly reduced intensity [42]. These results confirm that the aldehyde groups of ODex reacted with the amino groups of GelMA. The appearance of a characteristic imine bond peak at 1646 cm^−1^ in GM‐Odex further verified Schiff base formation. In the GM‐imid composite, the emergence of a C─S bond absorption peak at 740 cm^−1^ indicated successful grafting of 2‐mercapto‐1‐methylimidazole onto the GelMA backbone via thiol‐ene click chemistry [26].

Rheological analysis demonstrated that all groups formed stable gel structures after 1 min blue‐light irradiation, with storage modulus (*G*') consistently exceeding loss modulus (*G*''). Increasing GelMA concentration from 15% to 20% significantly enhanced the storage modulus. The introduction of ODex further improved the modulus through double‐network formation (Figure 2B). Notably, although the thiol groups in 2‐MMI consume part of the C═C bonds in GelMA, the chosen GelMA featured both a high degree of substitution (DS60) and a sufficiently high concentration, and the strong hydrogen‐bond coordination from imidazole groups enabled GM‐imid to exhibit superior mechanical properties compared to pure GelMA at the same concentration. Frequency sweep tests revealed that all hydrogels showed increasing loss modulus with rising angular frequency (Figure 2C), with GM‐Odex and GM‐imid demonstrating higher loss modulus inflection points, indicating superior shear deformation resistance.

The injectability of the hydrogels was examined to evaluate their surgical suitability. As shown in Figure 2D, non‐crosslinked GM‐Odex and GM‐imid@MPDA hydrogels could be smoothly extruded through a 27G syringe needle in PBS, forming continuous “WMU” patterns. Notably, even when applied sequentially, the previously crosslinked inner hydrogel formed a tight interface with the later‐applied outer hydrogel, greatly facilitating surgical manipulation. Considering the spatial constraints of the optic nerve region, the swelling behavior of the hydrogels was investigated. Pure GelMA hydrogels showed high swelling ratios, with 20% GelMA reaching 21.04 ± 1.65% (Figure S1). The introduction of ODex and 2‐MMI significantly reduced swelling, with G20OD5 and G20imid5 showing ratios as low as 10.68 ± 3.13% and 12.94 ± 2.08% (Figure 2E, F), respectively, effectively preventing secondary damage to the optic nerve caused by excessive swelling.

To better approximate the local CSF environment surrounding the optic nerve, a mi‐croinjection system was further applied to simulate slow fluid perfusion. Degradation times increased from approximately 120 h in 10% GelMA to 300 h in G20OD5 and G20imid5, primarily due to the dual‐network structure and the additional hydrogen bonding introduced. Early in the degradation process, the residual mass of some hydrogel samples briefly exceeded their initial mass, possibly attributed to a swelling rate that outpaced the degradation rate during the initial stage of collagenase digestion (Figure 2G, H and Figure S2). Tensile tests at 60% strain demonstrated that G20OD5 and G20imid5 displayed the highest tensile strength (113.01 ± 19.26 kPa and 97.85 ± 16.81 kPa, respectively) (Figure 2I), indicating excellent elasticity and deformation resistance capable of withstanding tissue traction and cerebrospinal fluid pressure.

Because the optic nerve is located in a deep anatomical position and is bathed in cerebrospinal fluid within its sheath, the surgical field remains constantly moist. To assess the adhesion performance of the outer hydrogel (GM‐Odex) under these wet conditions, a porcine optic nerve model was utilized (Figure 2J). Following fenestration of the optic nerve sheath, the GM‐Odex hydrogel was applied and crosslinked under PBS solution, demonstrating robust adhesion to the sheath incision (Figure 2K).

After curing, the hydrogel exhibited a characteristic pale‐yellow appearance and maintained stable adhesion even under fluid rinsing. A burst‐pressure setup was subsequently employed to evaluate the adhesive strength of the hydrogels In Vitro (Figure 2L). Among these, G15OD5 displayed the strongest sealing capability, tolerating liquid pressures exceeding 150 mmHg, while G20OD5, though slightly less robust, still with‐stood up to 100 mmHg (Figure 2M). Because the upper limit of normal adult cerebrospinal fluid pressure is approximately 18.4 mmHg, and optic‐nerve‐related subarachnoid pressure is considered to be within a broadly comparable range, the burst pressure sup‐ported by G20OD5 already provides a substantial safety margin above the expected physiological pressure environment. Meanwhile, compared with G15OD5, G20OD5 exhibited lower swelling, longer degradation time, and more favorable overall mechanical stability. Therefore, after balancing sealing capacity with swelling control, durability, and structural robustness, G20OD5 was selected as the outer layer formulation in the present study.

### 3.3 Synthesis and Characterization of MPDA‐Cle NPs

**FIGURE 3:**
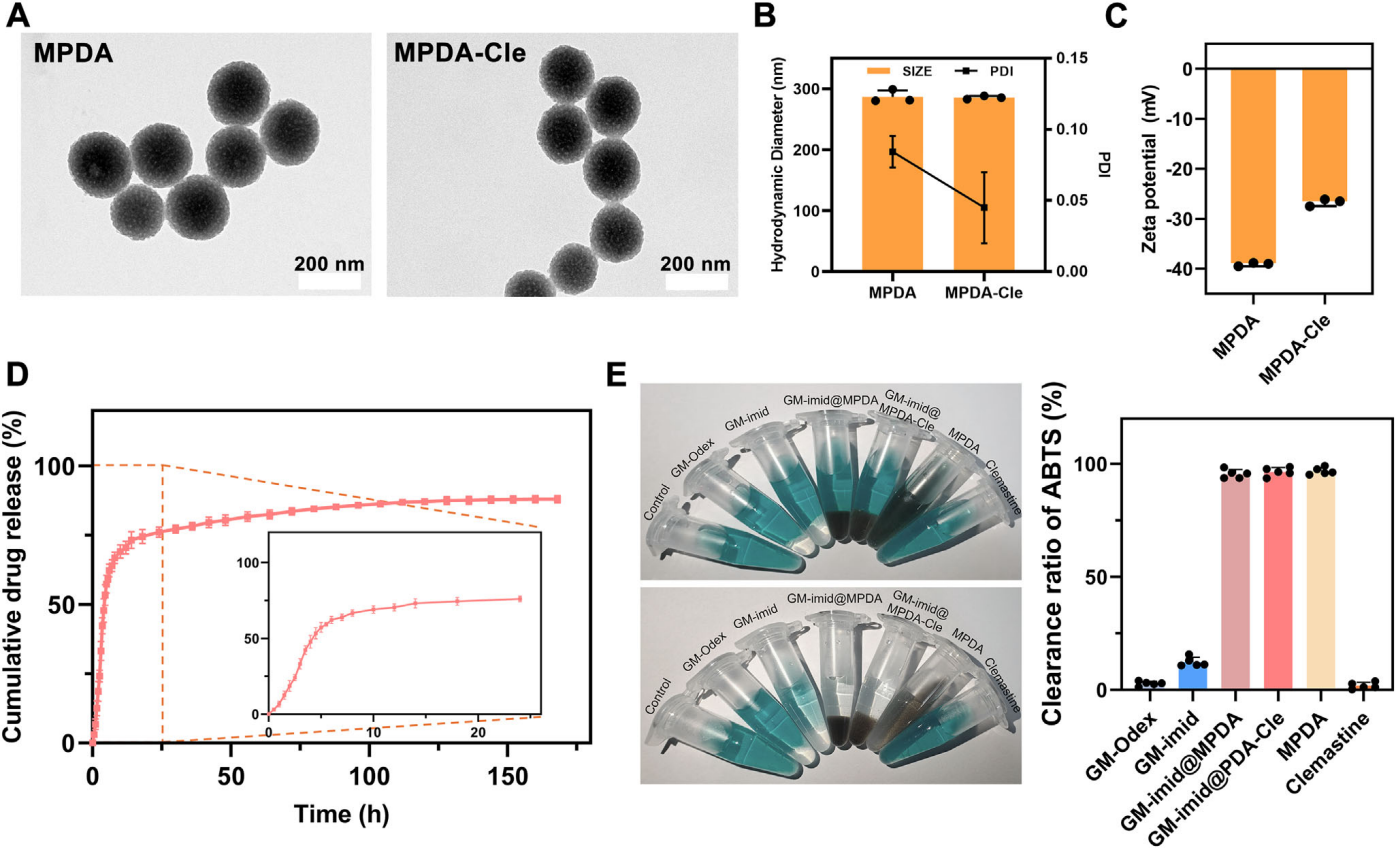
(A) TEM images of MPDA and MPDA‐Cle NPs (scale bars: 200 nm). (B) Hydrodynamic distribution and (C) Zeta potential of MPDA, MPDA‐Cle NPs (n = 3). (D) Cumulative release of clemastine from MPDA‐Cle nanodrug dispersion (n = 3). (E) Visual appearance and radical scavenging ratio of ABTS^•+^ after different treatments for 3 h (n = 5).

MPDA NPs were successfully synthesized via a template‐removal method under mild alkaline conditions [28]. After removing the F127 and TMB templates, the resulting MPDA NPs exhibited a regular spherical morphology with an average diameter of 183.7 ± 10.01 nm. Transmission electron microscopy (TEM) images revealed distinct nanoscale pores on the particle surface (Figure 3A). Combined with the nitrogen adsorption‐desorption measurements showing a specific surface area of 60.2 m^2^/g and a total pore volume of 0.367 cm^3^/g (Figure S3), these results confirm the structural advantages of MPDA NPs as drug carriers.

In this study, clemastine—an antihistamine recently reported to promote oligodendrocyte precursor cell differentiation and myelin regeneration via the muscarinic M1 receptor pathway [29, 43, 44]—was selected as a therapeutic agent. In commercial formulations, clemastine is typically used in its fumarate form to enhance stability. However, this salt form exhibits extremely poor water solubility, leading to severely limited bioavailability. To address this key technical challenge, clemastine was loaded into the mesoporous structure of MPDA NPs, thus constructing MPDA‐Cle nanomedicines. Dynamic light scattering (DLS) analysis revealed that the hydrodynamic diameters of MPDA NPs and MPDA‐Cle NPs were 286.9 ± 10.52 nm and 285.6 ± 2.71 nm, respectively, with polydispersity indices (PDIs) of 0.08 ± 0.01 and 0.04 ± 0.02 (Figure 3B). Notably, the decreased PDI after drug loading indicates that the spatial shielding effect of drug molecules and the optimized surface charge on MPDA NPs significantly improve nanoparticle dispersion stability. Zeta potential measurements showed that after drug loading, the potential increased from −39.1 ± 0.36 mV to −26.7 ± 0.72 mV (Figure 3C), further confirming successful drug encapsulation. Utilizing the mesoporous structure of MPDA NPs and multiple intermolecular forces, including hydrophobic interactions, hydrogen bonding, π‐π stacking, and electrostatic interactions, high‐efficiency loading of the hydrophobic clemastine fumarate was achieved, with a loading capacity of 15.18 ± 0.13%.

### 3.4 Sustained Release of clemastine

In Vitro release studies demonstrated that the MPDA‐Cle nanodrug provided sustained release of clemastine over one week (Figure 3D). This sustained‐release profile provides a prolonged local exposure window that may be relevant to oligodendrocyte‐associated maturation and myelin repair. Such a release pattern not only extends the therapeutic window for neuroprotection but also avoids potential side effects caused by fluctuations in blood drug levels, offering an ideal pharmacological strategy for optic nerve repair.

### 3.5 Free Radical Scavenging Activity

The antioxidant capacity of these materials was evaluated via ABTS radical‐scavenging assays (Figure 3E). The results revealed that components containing MPDA effectively scavenged ABTS^•+^ radicals, changing the solution from blue to colorless—a benefit primarily attributed to the intrinsic antioxidative properties of melanin‐like materials and the high specific surface area of MPDA [45]. MPDA‐containing formulations showed clear radical‐scavenging activity, whereas GM‐imid exhibited only very weak antioxidant activity, likely stemming from the reducing thiol group in 2‐MMI and the imidazole ring's conjugated system [46], and GM‐Odex showed no evident antioxidant activity under the present conditions. These findings underscore the dual advantages of the drug delivery system, combining robust sustained‐release capability with antioxidant functionality, and thus offering an innovative solution for treating optic nerve injuries accompanied by oxidative stress.

### 3.6 In Vitro Biocompatibility Evaluation and Intracellular ROS Scavenging Capacity

**FIGURE 4:**
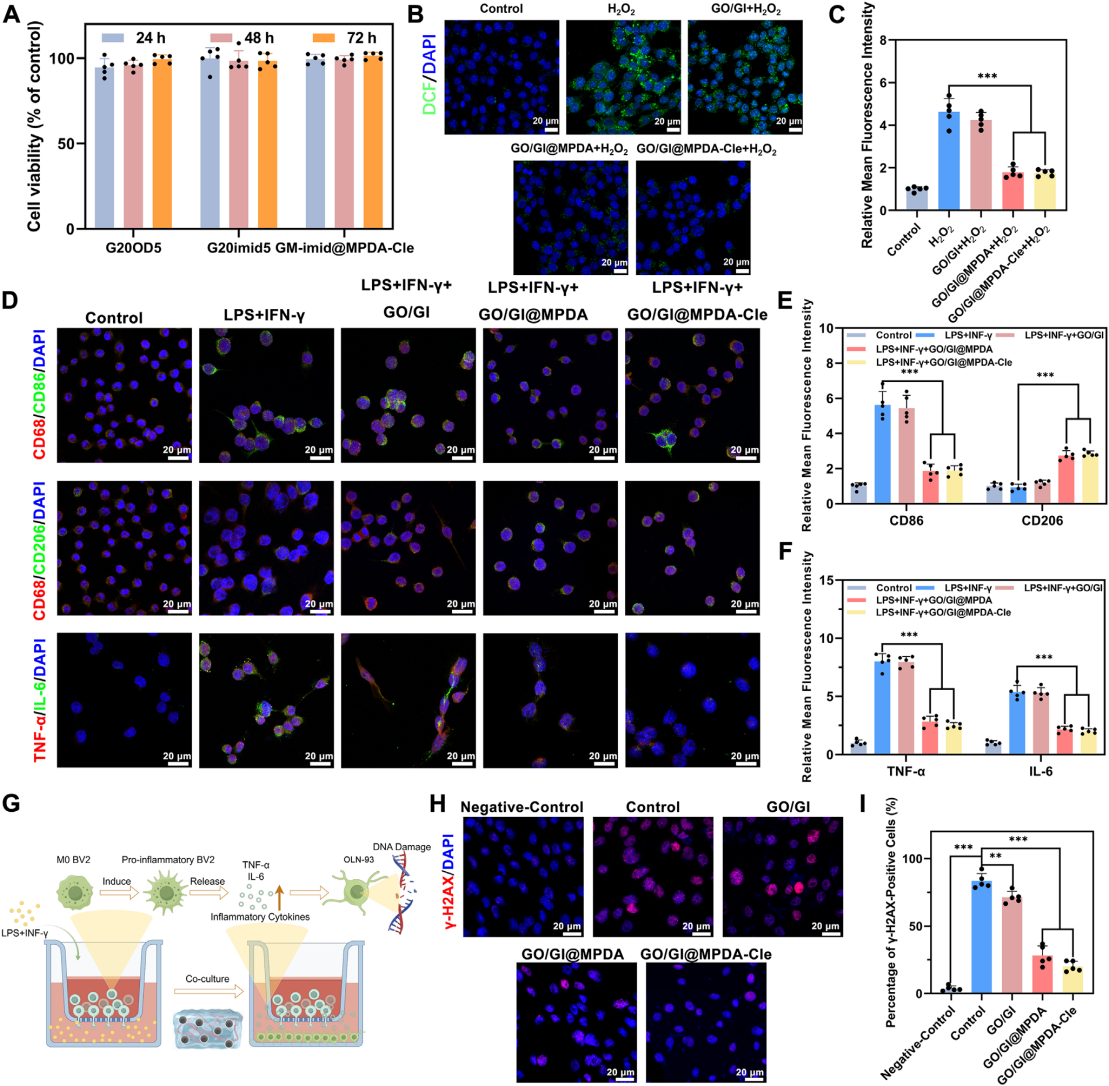
Evaluation of In Vitro Cytotoxicity, Antioxidant Properties, and Immunomodulatory Capacity of GO/GI@MPDA‐Cle. (A) Cytotoxicity effects of GM‐Odex, GM‐imid, and GO/GI@MPDA‐Cle hydrogels on R28 cells by CCK‐8 assay. (B) DCF fluorescence images and (C) mean fluorescence intensity of R28 cells upon various treatments. (D) Immunofluorescence images and (E, F) mean fluorescence intensity of BV2 cells staining for CD68, CD86, CD206, TNF‐α, and IL‐6 after different treatment. (G) Schematic diagram of oligodendrocyte (OLN‐93) DNA damage induced by co‐culture with pro‐inflammatory microglia (BV2 cells). (H) Representative immunofluorescence images and (I) quantification of γ‐H2AX mean fluorescence intensity in OLN‐93 cells after different treatments. *p < 0.05, **p < 0.01, ***p < 0.001.

R28 cells, a progenitor cell line derived from rat retina, exhibit robust proliferative capacity and multi‐lineage differentiation potential. They are commonly employed as an In Vitro surrogate model for retinal ganglion cells (RGCs), enabling the evaluation of biomaterials for retinal research [34]. In this study, R28 cells were utilized to mimic RGC behavior, and their compatibility with various hydrogels was examined using CCK‐8 assays and live/dead staining. The results showed that the inner hydrogel (GM‐imid), the outer hydrogel (GM‐Odex), and the dual‐layer drug‐loaded hydrogel (GO/GI@MPDA‐Cle) displayed no obvious cytotoxicity over 72 h, maintaining cell viability above 90% (Figure 4A). Live/dead staining further corroborated these findings (Figure S4).

Oxidative stress is a key driver of secondary pathological changes following central nervous system (CNS) injury, primarily by inducing excessive accumulation of reactive oxygen species (ROS), triggering neuroinflammation, and resulting in neuronal damage [47]. Herein, an In Vitro oxidative stress model was established by stimulating R28 cells with H_2_O_2_. As shown in Figure 4B‐C, treatment with 100 µM H_2_O_2_ significantly increased the intensity of green DCF fluorescence in R28 cells, reflecting a marked elevation in intracellular ROS levels. During the evaluation of antioxidant capacity, the imidazole‐containing hydrogel (GO/GI) exhibited no significant ROS‐scavenging effect. In contrast, both GO/GI@MPDA and GO/GI@MPDA‐Cle effectively reduced intracellular ROS accumulation owing to the strong antioxidative potential of MPDA.

### 3.7 In Vitro Immunomodulatory Capacity

In the context of optic nerve injury, activated microglia are major contributors of ROS and pro‐inflammatory mediators (e.g., TNF‐α, IL‐6) [48]. These factors further polarize microglia toward the pro‐inflammatory phenotype, forming a vicious cycle that exacerbates neurological dysfunction. To evaluate the impact of hydrogels on microglial phenotypic modulation, BV2 cells (a mouse microglial cell line) were induced to a proinflammatory state using LPS and IFN‐γ. As shown in Figure 4D–F, these stimuli markedly increased the expression of the pro‐inflammatory marker CD86, while the anti‐inflammatory marker CD206 was nearly undetectable in BV2 cells, and the TNF‐α‐ and IL‐6‐associated immunofluorescence signals were significantly increased. Compared with the control and GO/GI groups, both GO/GI@MPDA and GO/GI@MPDA‐Cle significantly downregulated CD86 expression, while upregulating that of CD206, accompanied by decreases in TNF‐α and IL‐6 levels. These results indicate that the hydrogels effectively shift BV2 cells from a pro‐inflammatory phenotype to an anti‐inflammatory phenotype.

To further examine whether this anti‐inflammatory effect was associated with NF‐κB‐ and inflammasome‐related signaling, additional Transwell‐based experiments were per‐formed in LPS‐induced microglia. As shown in Figures S5 and S6, GO/GI alone showed little effect, whereas MPDA, GO/GI@MPDA, and GO/GI@MPDA‐Cle alleviated P65/IκBα‐related activation and reduced the expression of NLRP3, IL‐1β, and caspase‐1. These supplementary results further support the view that the principal anti‐inflammatory contribution in the present system arises from MPDA.

Pro‐inflammatory microglia exert considerable toxicity on surrounding neurons and glial cells [48]. Given that oligodendrocyte‐derived myelin sheaths are crucial for optic nerve signal transmission, we further employed a Transwell coculture system (Figure 4G) to assess the effects of hydrogel‐treated BV2 cells on the oligodendrocyte cell line OLN‐93. The results indicated that co‐culturing OLN‐93 cells with proinflammatory BV2 cells induced significant DNA damage, as assessed by γ‐H2AX (Figure 4H, I). This DNA damage effect was significantly alleviated when OLN‐93 cells were co‐cultured with pro‐inflammatory BV2 cells that had been pre‐treated with GO/GI@MPDA or GO/GI@MPDA‐Cle. These findings demonstrate that the hydrogel effectively modulates microglial phenotypes, improves the deteriorated microenvironment, and thereby reducing inflammation‐associated injury in OLN‐93 cells.

In summary, the MPDA‐containing hydrogel formulations demonstrated marked an‐tioxidant and anti‐inflammatory activity, directly reducing ROS accumulation and pro‐moting microglial polarization toward a more neuroprotective phenotype, while also indirectly protecting myelin‐forming cells by improving the cellular microenvironment. These findings provide In Vitro experimental support for the potential of this bilayer platform as a multifunctional local strategy for optic nerve injury treatment.

### 3.8 In Vivo Biocompatibility Evaluation

**FIGURE 5:**
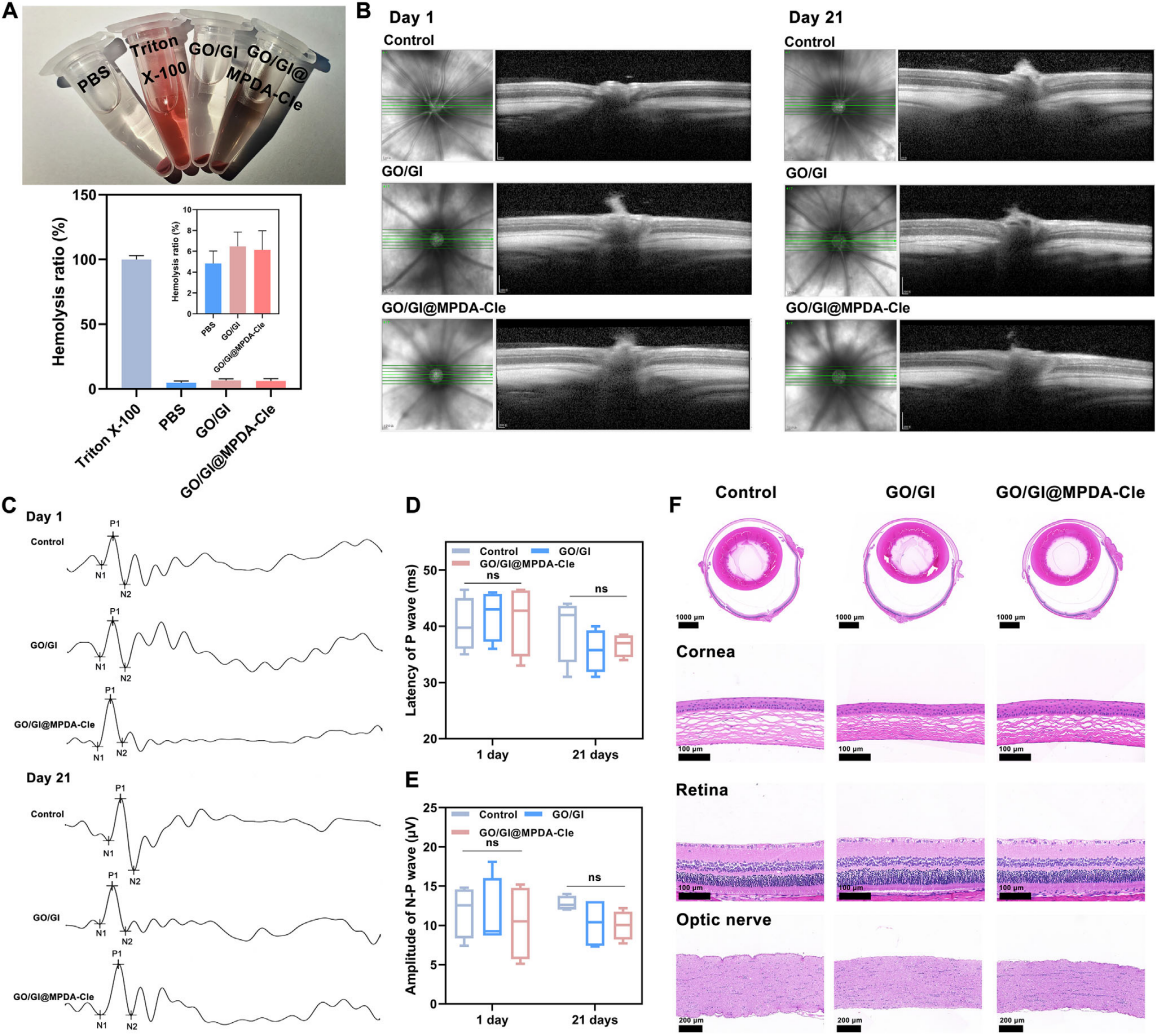
In Vivo biocompatibility evaluation of GO/GI@MPDA‐Cle. (A) Representative photographs and In Vitro hemolysis ratio of rat red blood cells after treatment with PBS, Triton X‐100, GO/GI, and GO/GI@MPDA‐Cle (n = 3). (B) OCT images of the optic disc at 1 and 21 days after local application of GO/GI or GO/GI@MPDA‐Cle in healthy rat optic nerves. (C) Representative fVEP traces and quantitative analysis of P‐wave latency (D) and N‐P wave amplitude (E). (F) H&E staining of the eyeball, cornea, retina, and optic nerve at 21 days post‐application. *p < 0.05, **p < 0.01, ***p < 0.001, and ns, no significance.

Good In Vivo biocompatibility is a prerequisite for the clinical application of biomaterials. For bilayer hydrogels intended for use in bodily fluids and blood environments, hemolytic properties are a key parameter for evaluating blood compatibility. In this study, rat red blood cells were used to assess the hemolytic activity of GO/GI and GO/GI@MPDA‐Cle. As shown in Figure 5A, cells treated with 0.1% Triton X‐100 showed severe hemolysis, while neither the GO/GI nor GO/GI@MPDA‐Cle groups caused significant hemolysis, indicating favorable hemocompatibility of both materials.

Since GO/GI@MPDA‐Cle is designed for local application in the optic nerve, its post‐implantation swelling behavior and degradation products could potentially affect the structure and function of the retina and optic nerve. To evaluate its In Vivo structural safety, in situ and noninvasive examinations were conducted using optic disc optical coherence tomography (OCT). OCT is a high‐resolution, cross‐sectional imaging technique that clearly reveals the layered structure of the retina. As shown in Figure 5B, on the first day after implantation, mild retinal edema was observed in all groups; however, no significant differences were found among them, possibly due to minor traction on the optic nerve during surgery. By day 21, retinal edema had subsided in all groups, retinal layers were well‐defined, and no pathological changes were detected, indicating that neither GO/GI nor GO/GI@MPDA‐Cle caused noticeable retinal structural damage when locally applied to the optic nerve.

To further assess the impact of the hydrogels on optic nerve function, flash visual evoked potentials (fVEP) were recorded. fVEP is an objective electrophysiological indicator of visual pathway function, where the P‐wave latency reflects nerve conduction speed, and the N‐P wave amplitude represents the intensity of synchronized firing in the optic nerve [49]. As shown in Figure 5C–E, fVEP waveforms were generally consistent across groups, with no statistically significant differences in P‐wave latency or N‐P wave amplitude. These findings indicate that the implantation of GO/GI and GO/GI@MPDA‐Cle did not produce observable adverse effects on visual signal conduction. On day 21 post‐operation, hematoxylin‐eosin (H&E) staining was performed to evaluate the overall ocular structure. As shown in Figure 5F, the cornea, retina, and optic nerve in all groups remained intact, with no inflammatory cell infiltration or pathological changes observed, thereby confirming the local biocompatibility of the materials at the histological level.

Collectively, the In Vivo results demonstrate that the GO/GI@MPDA‐Cle hydrogel possesses excellent blood compatibility and local tissue compatibility. When implanted around the optic nerve, it did not induce significant hemolysis, retinal structural damage, visual functional impairment, or histopathological alterations, indicating superior In Vivo safety. These findings provide a reliable foundation for its potential use as a long‐term drug carrier in the field of optic nerve protection.

### 3.9 Establishment of the Partial Optic Nerve Injury (PONI) Model and Assessment of GO/GI‐Mediated Local Drug Delivery

**FIGURE 6:**
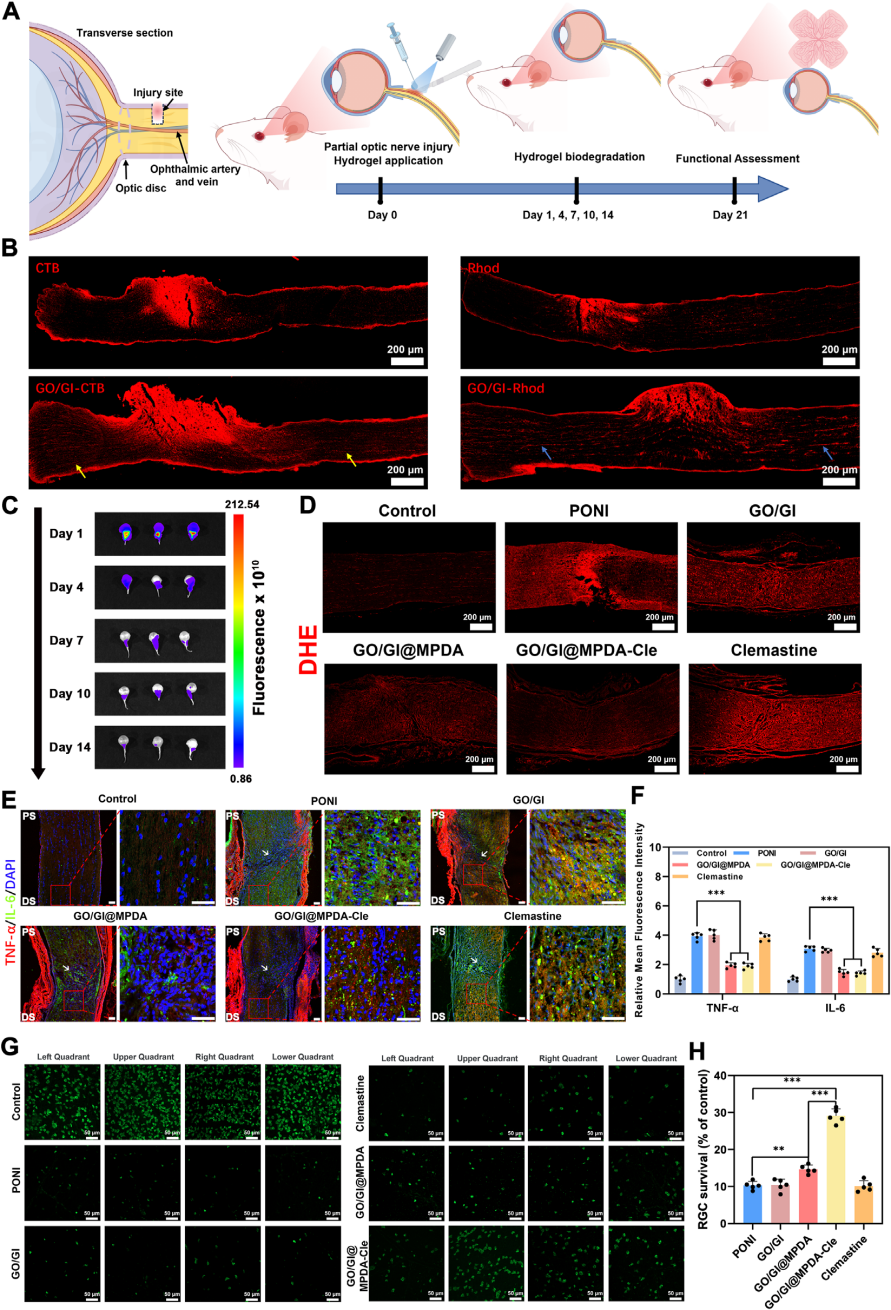
In Vivo application of GO/GI@MPDA‐Cle modulates the immune microenvironment and protects RGCs in PONI model. (A) Schematic diagram of PONI and the corresponding animal experimental flow chart. (B) Fluorescence images of optic nerve sections 3 days after different treatments (The yellow arrows indicate axons labeled by CTB, and the blue arrows indicate rhodamine‐positive signals within the optic nerve tissue. scale bars: 200 µm). (C) Ex vivo fluorescence images of the eyeball and optic nerve tissues at various time points after local application of GO/GI‐Rhod. (D) DHE staining of optic nerves at 3 weeks post‐PONI under different treatments (scale bars: 200 µm). (E) Immunofluorescence staining of TNF‐α and IL‐6 and (F) corresponding mean fluorescence intensity at 3 weeks post‐PONI under different treatments (scale bar: 50 µm). (G) Representative retinal flat‐mount images (labeled with Brn3a) from four quadrants at 3 weeks post‐PONI under different treatments, and (H) the statistical analysis of surviving RGC proportions. *p < 0.05, **p < 0.01, ***p < 0.001.

Although the optic nerve crush model is commonly used to investigate traumatic optic neuropathy, it leads to complete transection of axons, which differs from the partial injury commonly observed in clinical practice. Clinically, most patients present with partial optic nerve injury, accompanied by a persistent deterioration of the local microenvironment that ultimately causes secondary neuronal death, axonal demyelination, and atrophy [13, 35]. Therefore, this study employed a PONI model that more closely resembles clinical pathological features to evaluate the local therapeutic efficacy of GO/GI@MPDA‐Cle. Specifically, as shown in Figure 6A, the optic nerve was half‐cut 2 mm behind the eyeball and immediately treated with various hydrogels at the injury site. Relevant parameters were then assessed, and tissue samples were collected on postoperative days 1, 4, 7, 10, 14, and 21. To verify the successful establishment of the PONI model, cholera toxin subunit B (CTB), an axonal tracer, was used to assess the extent of partial axonal transection. As shown in Figure S7, 21 days post‐operation, the CTB‐labeled axons were interrupted at the injury site (red arrow), and only about half of the optic nerve area (demarcated by the yellow dotted line) was effectively labeled. Meanwhile, the retinal flat‐mount results (Figure S8) revealed that, compared with the sham group, the PONI group displayed extensive RGC loss across the whole retina at 21 days post‐injury, with more pronounced loss in the half of the retina directly injured. This discrepancy arises from primary RGC death on the injured side due to direct axonal transection, whereas secondary RGC death on the contralateral side is mainly attributed to a deteriorating microenvironment. Collectively, these findings confirm the reliability of the PONI model.

To investigate the local drug delivery capability of the double‐layer hydrogel, CTB and rhodamine were respectively loaded into the GO/GI hydrogel. Because MPDA particles exhibit a broad‐spectrum light absorption that interferes severely with fluorescence imaging, they were excluded from the carrier system in this experiment. As shown in Figure 6B, compared with traditional local injection, CTB in the GO/GI‐CTB group was released from the hydrogel and diffused along both sides of the optic nerve, effectively labeling axons along the pathway (yellow arrows). Rhodamine released from the GO/GI‐Rhod hydrogel was detected within the surrounding optic nerve tissue, indicating local dispersion of the fluorescent tracer (blue arrows).

Small‐animal Ex vivo fluorescence imaging system was further used to monitor the retention of the rhodamine‐loaded hydrogel (GO/GI‐Rhod) at the optic nerve. As shown in Figure 6C and Figure S9, although the fluorescence signal in the optic nerve region gradually decreased over time, residual signal remained detectable within approximately 2 weeks, whereas by the third week no obvious hydrogel‐related signal could be identified.

In addition, fluorescence microscopy was used to examine the In Vivo retention and degradation behavior of the locally applied bilayer hydrogel. Residual fluorescence remained detectable at the lesion site at approximately 2 weeks, but gradually became difficult to observe thereafter, suggesting progressive local degradation and clearance of the system over time (Figure S10). The white polygon indicates the region of incompletely de‐graded bilayer hydrogel. These observations suggest that the hydrogel can remain locally over 2 weeks and may therefore serve as a transient depot to support sustained local delivery and microenvironmental regulation.

### 3.10 GO/GI@MPDA‐Cle Protects RGCs After Partial Optic Nerve Injury (PONI) by Regulating the Immune Microenvironment

Subsequently, the antioxidative and anti‐inflammatory effects of GO/GI@MPDA‐Cle at the injury site were evaluated. As shown in Figure 6D, at 3 weeks post‐injury, strong DHE fluorescence was observed at both the proximal (eye) and distal (brain) segment of the optic nerve in the PONI group, indicating severe ROS accumulation. Neither the local injection of clemastine nor the application of GO/GI hydrogel improved this situation, whereas GO/GI@MPDA and GO/GI@MPDA‐Cle treatments significantly reduced DHE fluorescence intensity, demonstrating effective ROS clearance at the injury site. Regarding inflammatory mediators, the injury triggered a significant increase in TNF‐α and IL‐6 (Figure 6E, F). However, both GO/GI@MPDA and GO/GI@MPDA‐Cle treatments effectively suppressed their expression, consistent with In Vitro findings.

The RGC survival rate at 3 weeks post‐injury was quantified using retinal flat‐mounts stained with Brn3a, a specific RGC marker (a POU‐domain transcription factor stably expressed in mature RGCs). As shown in Figure 6G, H, 3 weeks after PONI, the RGC survival rate dropped sharply to 10.28 ± 1.10%. Neither the clemastine group (10.37 ± 1.60%) nor the GO/GI group (10.11 ± 1.48%) exhibited marked improvement. Treatment with GO/GI@MPDA increased the RGC survival rate to 14.66 ± 1.15%, whereas GO/GI@MPDA‐Cle demonstrated the best protective effect, reaching 29.08 ± 1.93%. This synergistic neuroprotective effect was partly attributed to the MPDA component, which effectively modulated oxidative stress and neuroinflammation, and may also be related to the reported promyelinating activity of clemastine in experimental and clinical demyelinating conditions [29, 31, 43, 44]. Consequently, persistent activation of microglia by myelin debris was reduced, and secondary axonal atrophy caused by demyelination was prevented. Based on these findings, GO/GI@MPDA‐Cle showed a significant synergistic neuroprotective effect following PONI.

### 3.11 GO/GI@MPDA‐Cle Supports Optic Nerve Repair Through Myelin Preservation and Remyelination‐Associated Repair

Myelin disintegration following central nervous system injury is a complex pathological process, resulting not only from chronic demyelination that renders axons vulnerable but also from rapid oligodendrocyte loss leading to failed endogenous remyelination. Successful myelin stabilization and regeneration rely on a healthy microenvironment, in which properly regulated microglia play a pivotal role [50, 51, 52]. However, inflammatory responses and oxidative stress can disrupt this delicate process [53, 54]. Clemastine, an FDA‐approved antihistamine medication, has recently been found to effectively promote oligodendrocyte precursor cell (OPC) differentiation into mature oligodendrocytes by antagonizing muscarinic acetylcholine receptor M1R, thereby stimulating remyelination and protecting myelin integrity [30, 31, 43, 55]. In light of previous findings indicating that GO/GI@MPDA‐Cle exhibits excellent immunomodulatory capacity, further investigation was conducted to evaluate its protective and pro‐regenerative effects on myelin sheaths in a partial optic nerve injury model.

**FIGURE 7:**
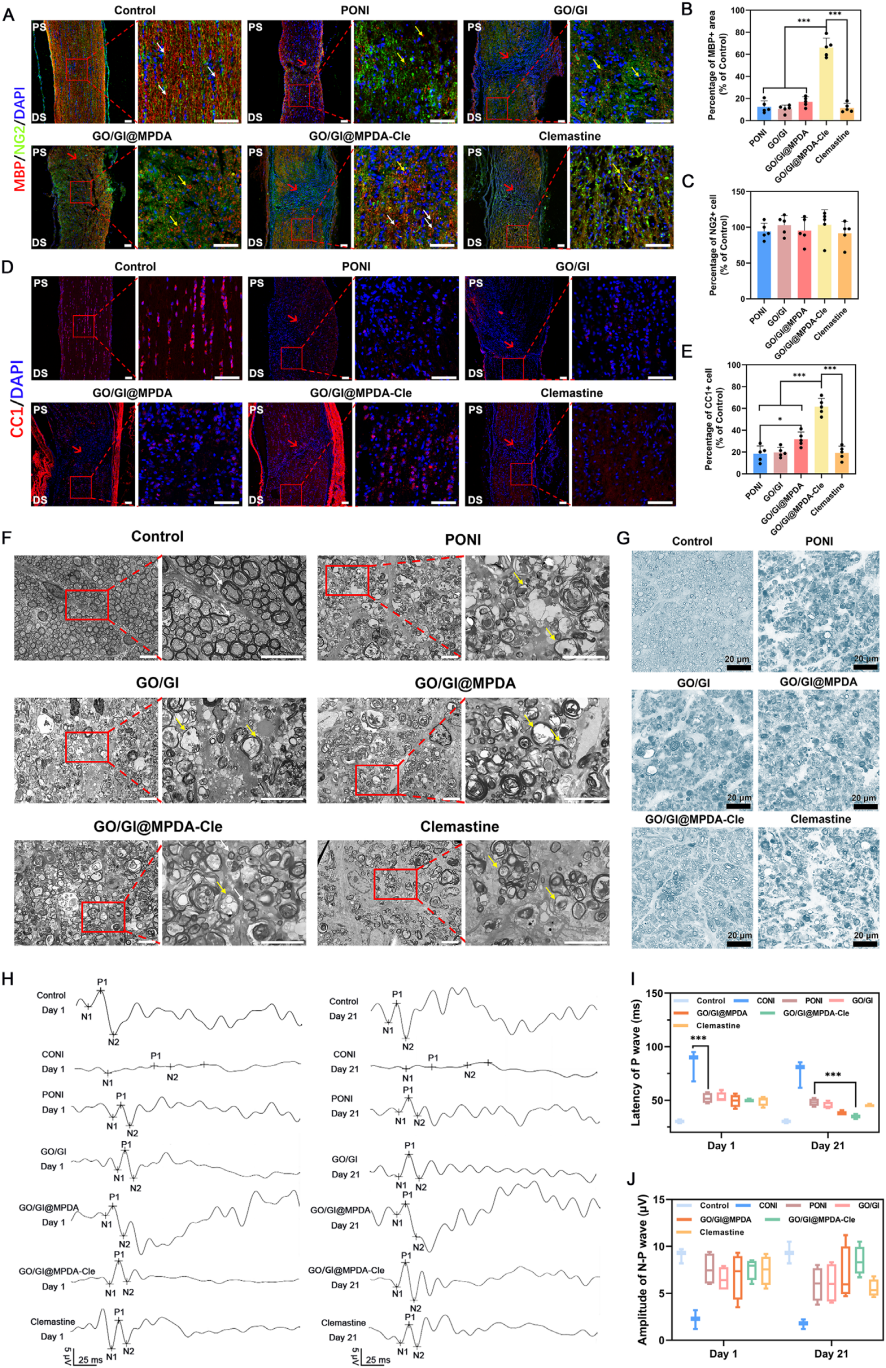
Myelin preservation, remyelination‐associated repair, and functional recovery assessment. (A) Representative immunofluorescence images of MBP and NG2 staining at 3 weeks post‐PONI under different treatments (scale bar: 50 µm). (B) Relative mean fluorescence intensity of MBP. (C) Quantification of NG2‐positive cells. (D) Representative immunofluorescence images of CC1 staining at 3 weeks post‐PONI under different treatments (scale bar: 50 µm). (E) Quantification of CC1‐positive cells. (F) TEM images of optic nerve cross‐sections at 3 weeks post‐PONI under different treatments (scale bar: 5 µm). (G) Toluidine blue staining of optic nerve cross‐sections at 3 weeks post‐PONI under different treatments (scale bar: 20 µm). (H) fVEP waveforms at 1 and 21 days post‐PONI under different treatments, with quantitative analysis of (I) P‐wave latency and (J) N‐P wave amplitude. The white arrows indicate relatively preserved and compact myelin sheaths, whereas the yellow arrows indicate injured myelin profiles showing swelling, vacuolization, discontinuity, or disintegration. *p < 0.05, **p < 0.01, ***p < 0.001.

Myelin basic protein (MBP) is a major structural component of myelin and is commonly used to assess myelin‐associated structure and integrity. However, MBP staining alone cannot strictly distinguish between residual myelin, damaged myelin fragments, and newly formed myelin sheaths. As shown in Figure 7A, B, MBP‐associated signals displayed a relatively continuous and regular distribution in normal optic nerves. At 3 weeks post‐injury, the signals were markedly reduced and showed fragmented, punctate, or annular patterns, consistent with reduced myelin‐associated structure and increased myelin fragmentation. In contrast, GO/GI@MPDA‐Cle treatment improved the continuity and integrity of the MBP‐associated signal. To investigate the underlying mechanisms, the expression of the OPC marker NG2 and the mature oligodendrocyte marker CC1 was assessed [56, 57]. As presented in Figure 7C, there was no significant difference among the groups in the proportion of NG2‐positive cells, indicating that NG2‐positive cells remained detectable within the injured optic nerve at this time point. These findings suggest that the myelin‐associated changes observed at this stage were not simply attributable to the absence of NG2‐positive cells. Consistent with this, CC1 immunostaining (Figure 7D, E) revealed a marked decline in the number of mature oligodendrocytes 3 weeks post‐injury. Neither GO/GI nor clemastine alone improved this situation, while GO/GI@MPDA partially maintained CC1‐positive cell numbers, likely attributable to the MPDA component's positive modulation of the microenvironment. Notably, GO/GI@MPDA‐Cle showed the most pronounced increase in CC1‐positive cell numbers among the treatment groups. These findings are consistent with a potential role of the composite system in preserving and supporting the maturation of oligodendrocyte‐associated cells through local microenvironmental regulation and sustained clemastine delivery.

To further evaluate the morphology of myelin sheaths at the ultrastructural level, transmission electron microscopy (TEM) was performed. As shown in Figure 7F, normal optic nerves exhibit uniformly dense myelin sheaths tightly wrapped around axons (white arrows). In contrast, the PONI group displayed a marked reduction in myelin coverage, and the remaining myelin sheaths contained numerous vacuolated structures (yellow arrows), suggesting swelling and disintegration. In the GO/GI@MPDA‐Cle group, however, myelin ultrastructure was better preserved. Higher‐magnification TEM images and additional morphometric analyses, including the percentage of myelinated axons and the g‐ratio, are provided in Figure S11A–C. These morphological findings support myelin preservation but cannot by themselves distinguish residual myelin from newly formed myelin. Toluidine blue‐stained sections provided complementary structural observations over a broader field of view (Figure 7G and Figure S12).

Finally, the physiological significance of myelin improvement was assessed using functional indicators. Flash visual evoked potential (fVEP) recordings (Figure 7H–J) showed significantly prolonged P‐wave latency in all experimental groups compared to the control group at 1 day post‐injury. By postoperative day 21, the GO/GI@MPDA‐Cle group showed a significant reduction in P‐wave latency compared with the PONI group, consistent with partial improvement in optic nerve conduction‐related function. The N‐P wave amplitude showed an upward trend in the GO/GI@MPDA‐Cle group, but the difference did not reach statistical significance. To further assess visual pathway function at a later stage, the pupillary light reflex was evaluated at 8 weeks post‐PONI. This time point was selected because the residual optic nerve structure and function in the untreated PONI group had undergone pronounced progressive deterioration, providing a more suitable window to examine whether treatment could preserve residual visual pathway function. The GO/GI@MPDA‐Cle group showed a significantly greater pupillary constriction response than the PONI group, providing complementary physiological evidence consistent with partial improvement in visual pathway‐related function (Figure S13).

Taken together, these results demonstrate that GO/GI@MPDA‐Cle treatment was associated with reduced myelin loss, improved preservation of myelin‐associated structures, and increased numbers of CC1‐positive cells. These findings are consistent with oligodendrocyte‐associated maturation and remyelination‐associated repair, and were accompanied by partial improvement in optic nerve conduction‐related function.

### 3.12 Transcriptomic Profiling Reveals Multi‐Target Mechanisms of GO/GI@MPDA‐Cle in Optic Nerve Protection

**FIGURE 8:**
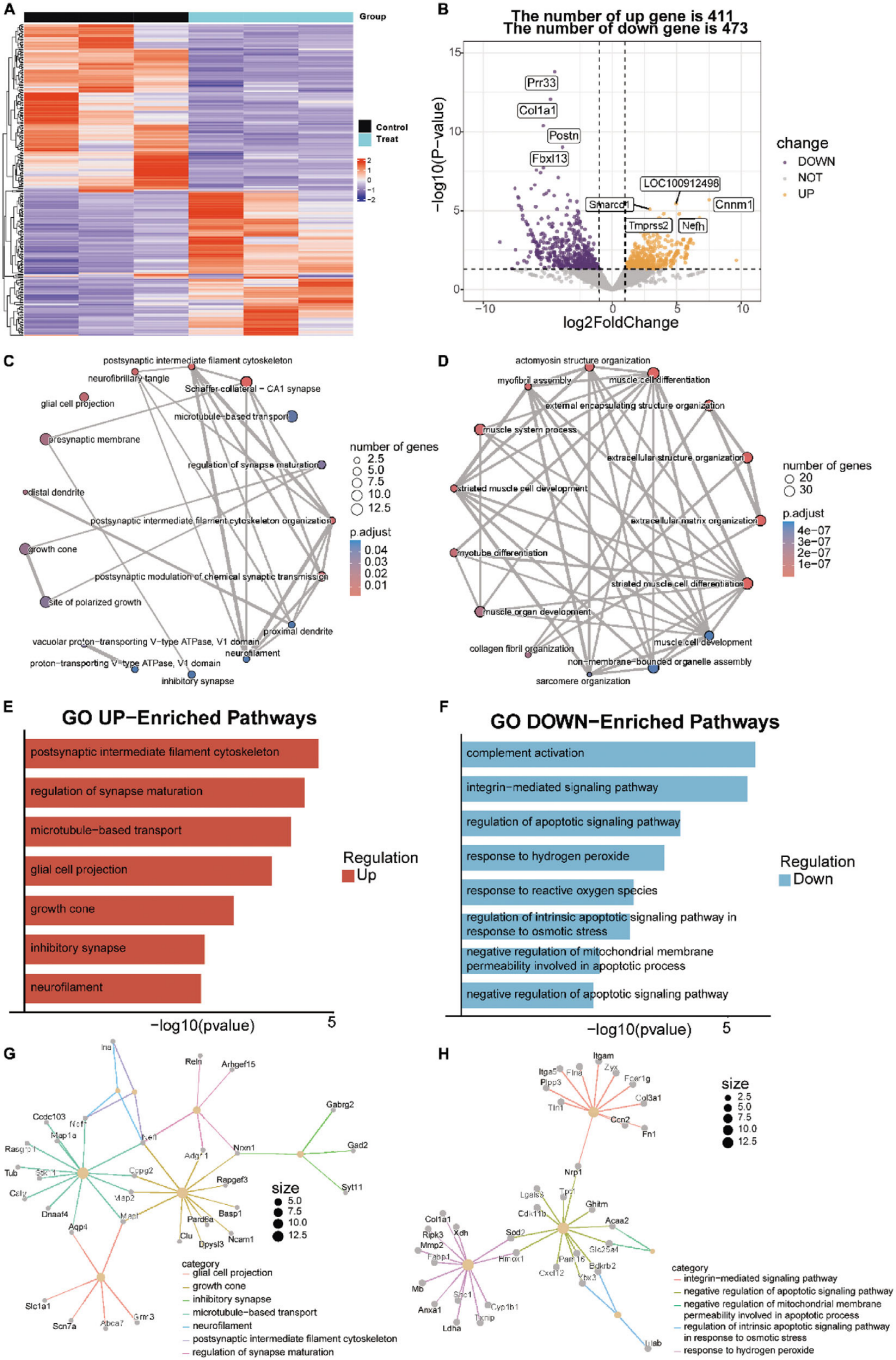
Transcriptomic analysis of the optic nerve 3 weeks post‐PONI following GO/GI@MPDA‐Cle hydrogel application. (A) Heatmap of differentially expressed genes (DEGs) between the PONI group and the GO/GI@MPDA‐Cle‐treated group after filtering out genes with high intra‐group variability (n = 3). (B) Volcano plot of DEGs. Bubble plots of the top enriched GO terms for (C) up‐regulated and (D) down‐regulated differentially expressed genes (DEGs). (E, F) Bar plots of the selected enriched GO terms for DEGs. (G, H) Network diagrams of key intersection genes.

Transcriptomics uses high‐throughput sequencing to comprehensively profile all RNA molecules in specific cells or tissues under defined conditions, revealing global regulatory patterns of gene expression [58]. To further validate the multi‐target regulatory effects of GO/GI@MPDA‐Cle on the lesional microenvironment at the molecular level, we performed transcriptome sequencing of the injured optic nerve regions in the PONI model and compared the GO/GI@MPDA‐Cle‐treated group with untreated PONI controls. Transcriptomic profiling demonstrates that GO/GI@MPDA‐Cle treatment induces significant gene expression alterations, where the heatmap (Figure 8A) visually represents global expression disparities between groups through red and blue color gradients, with red regions indicating up‐regulated genes and blue regions indicating down‐regulated genes. The volcano plot (Figure 8B) identifies 411 significantly up‐regulated genes and 473 down‐regulated genes based on stringent thresholds (|log_2_FC| > 1 and adjusted p value (padj) < 0.05), with each dot position precisely reflecting fold‐change magnitude and statistical significance.

GO enrichment analysis confirms the multi‐target reprogramming of the lesion microenvironment by GO/GI@MPDA‐Cle. Up‐regulated DEGs are mainly enriched in neuron structure and synaptic plasticity‐related terms, including postsynaptic intermediate filament cytoskeleton, Schaffer collateral‐CA1 synapse, regulation of synapse maturation, and growth cone [59, 60] (Figure 8C). Up‐regulation of genes such as *NEFL*, *MAP2*, and *SYN1* likely promotes stabilization of dendrite‐axon cytoskeletons, synapse maturation, and synaptic vesicle regulation, thereby supporting RGC axonal protection and functional recovery. In contrast, down‐regulated DEGs are significantly enriched in extracellular matrix organization, collagen fibril organization, actomyosin structure organization, and muscle system process [61, 62] (Figure 8D), accompanied by reduced expression of *COL1A1*, *FN1*, and *TNC*, consistent with attenuated fibrosis and contractile scarring that would otherwise exacerbate mechanical compression and secondary damage.

RNA sequencing analysis confirmed at the transcriptional level that GO/GI@MPDA‐Cle effectively reprograms the harmful injury microenvironment into a repair‐conducive state. The enrichment of up‐regulated DEGs in key neurorepair processes including neuronal structure and synaptic plasticity‐related processes (such as postsynaptic intermediate filament cytoskeleton, regulation of synapse maturation, growth cone) and glial cell‐related structural dynamics (such as glial cell projection) strongly suggests promotion of synaptic integrity, axonal growth, and oligodendrocyte‐mediated support functions (Figure 8E). Concurrently, significant down‐regulation of DEGs involved in complement activation, integrin‐mediated signaling, response to reactive oxygen species/hydrogen peroxide, and regulation of apoptotic signaling pathways (Figure 8F) molecularly corroborates the alleviation of neuroinflammation, oxidative stress, and neuronal apoptosis observed in our functional experiments.

Network analysis of transcriptomic data revealed cytoskeletal genes such as *NEFL*, *NEFH*, *MAP1A*, *MAP2*, and *MAPT*, together with synapse‐associated genes *RAPGEF3*, *NRXN1*, and *GABRG2*, indicate reinforced axonal scaffolding, growth‐cone dynamics, and refined inhibitory synaptic regulation, thereby supporting remyelination and restoration of synaptic plasticity (Figure 8G). Reduced expression of fibrosis/adhesion‐related molecules *FN1*, *COL1A1*, *ITGAM*, and *TNC* is consistent with attenuated scar formation, while the relative up‐regulation of antioxidant and anti‐apoptotic genes *SOD2*, *HMOX1*, *PAM16*, and *CXCL12* within these categories points to alleviated oxidative stress and apoptotic signaling (Figure 8H). Together, these network‐level changes substantiate that the hydrogel promotes neuronal survival and functional reconstruction via coordinated modulation of oxidative stress, apoptosis, and structural‐synaptic remodeling.

## 4 Discussion

Repair after optic nerve injury is not a single regenerative event, but a coupled process involving local barrier reconstruction, attenuation of inflammation and oxidative stress, support of remyelination, and preservation of residual conduction. The present study sought to integrate these interrelated but often separately addressed processes into a single local therapeutic system. Unlike conventional strategies that mainly emphasize either drug delivery or tissue sealing, this bilayer hydrogel design uses the outer layer for wet‐tissue adhesion and sheath closure, while the inner layer forms a non‐adhesive therapeutic interface for sustained MPDA‐Cle release. In this way, the system combines physical reconstruction with local microenvironmental intervention in the unique cerebrospinal‐fluid‐associated environment around the optic nerve. This design also suggests that, in central nervous system repair, biomaterials may act not only as drug carriers, but also as local organizers that help create conditions more permissive for residual tissue preservation and endogenous repair.

From a biological perspective, the present findings are more consistent with enhanced lesion‐site support and remyelination‐associated repair than with direct induction of long‐distance axonal regeneration across the injury. Remyelination is important not only for restoration of myelin structure, but also for maintenance of axonal integrity and recovery of effective conduction [55]. Whether this process proceeds successfully depends to a considerable extent on whether the post‐injury environment remains permissive for further differentiation and maturation of the oligodendrocyte lineage. At the same time, microglia‐associated inflammatory states continue to shape this process [51, 54]. In the present study, GO/GI@MPDA‐Cle was associated with reduced oxidative stress, attenuation of inflammatory responses, promotion of oligodendrocyte maturation, and preservation of myelin‐related structure. However, the present dataset does not allow a definitive distinction between preservation of residual myelin and de novo remyelination. Such a distinction would require dedicated oligodendrocyte‐lineage tracing, cell birth‐dating, or other longitudinal labeling strategies in combination with myelin markers. Within the current experimental framework, the findings therefore support myelin preservation and remyelination‐associated repair rather than direct proof of newly formed myelin sheaths. Taken together, these findings fit more closely with a model in which repair improves as the local environment is shifted toward a more supportive state.

The current In Vitro data also suggest that the principal anti‐inflammatory and antioxidant contribution arises from MPDA‐containing formulations, whereas the GM‐imid layer alone showed only very weak chemical radical‐scavenging activity and no clear cell‐level anti‐inflammatory effect in the present system. Within such a framework, the antioxidant and immunomodulatory effects of MPDA and the pro‐differentiation and promyelinating activity of clemastine may act on distinct but biologically coupled aspects of the lesion response [29, 43, 44]. In the acute PONI setting, sustained local delivery may be particularly relevant because it can prolong clemastine exposure at the lesion site while MPDA helps modulate the inflammatory and oxidative environment. For optic nerve disorders, this interpretation is also clinically relevant, because when stable long‐distance functional axonal regeneration remains difficult to achieve, preservation of residual neural structure and maintenance of residual conduction are important therapeutic objectives in their own right.

Another important feature of this system is that it is not an abstract material combination, but a structured design tailored to the local anatomy and surgical requirements of the optic nerve. The optic nerve is deeply located, the operative space is limited, and sheath fenestration requires both local closure and preservation of subarachnoid space patency. If a strongly adhesive single‐layer material were used alone, it could potentially interfere with cerebrospinal fluid flow on the subarachnoid side; conversely, a non‐adhesive single‐layer material would be unlikely to form a stable seal in the wet local environment. Thus, the significance of the bilayer structure is not simply the addition of another material layer, but the separation of the sealing function from the therapeutic interface so that each can serve a distinct local requirement. At the same time, both bilayer precursors are injectable and can be rapidly crosslinked under 405 nm blue‐light irradiation, which may make the platform compatible with endoscopic or other minimally invasive delivery approaches [63]. At present, optic nerve decompression can be performed through a transnasal endoscopic approach, and such systems are well suited to deep, narrow, and anatomically constrained regions. They may therefore provide a technical basis for future local material delivery and directed illumination. Because optic nerve sheath fenestration is usually limited in size, and the locally applied volume of bilayer precursor in this study is also small, effective coverage of the target window with endoscopic or directional blue‐light assistance is, in principle, feasible. In addition, the limited tissue penetration of blue light may help reduce the possibility of complete in situ gelation in non‐target regions. Combined with the local retention results, the system appears able to remain at the lesion site over a finite but therapeutically meaningful time window before gradually degrading and disappearing. This behavior, in which the material first acts locally and then gradually exits, is broadly consistent with the spatial‐control and safety requirements of local optic nerve therapy.

This study nevertheless remains at a proof‐of‐concept stage. The acute PONI model is suitable for testing whether the system can achieve local sealing, drug delivery, and microenvironmental modulation, but it does not fully recapitulate the long‐term pressure‐related injury, chronic inflammation, persistent demyelination, and glial scar formation seen in chronic optic neuropathies such as glaucoma. The present results also do not yet fully define long‐term adhesive stability under pulsatile cerebrospinal fluid conditions, the final fate of MPDA‐related products, or longer‐term visual outcomes. The functional assessments showed partial improvement in selected measures, including reduced P‐wave latency at 21 days and enhanced pupillary constriction at 8 weeks. However, the N‐P wave amplitude did not show a statistically significant recovery at 21 days. These findings provide complementary evidence of functional benefit, but do not establish complete restoration of optic nerve conduction or visual function. Future work will need to use models that better approximate clinical disease courses and combine longer‐term behavioral assessment, material‐fate analysis, dynamic cerebrospinal‐fluid simulation, delivery and illumination systems closer to actual surgical conditions. These studies will be important for determining the sustained effects of this platform on myelin preservation, remyelination‐associated repair, structural protection, and functional preservation in delayed‐treatment and chronic optic neuropathy models. Overall, this work suggests that local physical reconstruction, lesion microenvironment modulation, and remyelination support may be best understood as interdependent elements of optic nerve repair, and that a bilayer local hydrogel platform provides a feasible material framework for such coordinated intervention.

## Author Contributions

**Tonghe Pan**: Conceptualization, Methodology, Investigation, Writing – original draft. **Yate Huang**: Methodology, Investigation. **Junhao Zhang**: Methodology, Investigation. **Shudong Tian**: Methodology, Investigation. **Zhixing Li**: Methodology. **Xuanwen Li**: Investigation. **Yanchen Zhang**: Investigation. **Dongmei Wang**: Investigation. **Sihao Ye**: Investigation. **Ming Jin**: Investigation. **Neng Ling**: Investigation. **Wentao Yan**: Supervision, Resources, Project administration. **Kaihui Nan**: Resources, Supervision, Writing – review & editing. **Wencan Wu**: Writing – review & editing, Funding acquisition, Validation, Conceptualization, Resources, Supervision, Project administration. All authors have read and agreed to the published version of the manuscript.

## Funding

This work was financially supported by the National Key R&D Program of China (2021YFA1101200), the National Key R&D Program of China (2018YFA0701703), the National Natural Science Foundation of China (82171048), and the Key Science and Technology Program of Wenzhou (ZY2022021).

## Ethics Statement

The animal experiments were conducted with the Institutional Animal Care and Use Committee of the Eye Hospital of Wenzhou Medical University (Approval No. YSG23121401).

## Consent

The authors have nothing to report.

## Conflicts of Interest

The authors declare no conflicts of interest.

## Supporting information




**Supporting File**: advs78031‐sup‐0001‐SuppMat.docx.

## Data Availability

All data generated or analyzed during this work are included in this article and its supplementary information files. The data of this work is available from the corresponding authors on reasonable request.
