# Supplementary material for: In Situ Photo‑Crosslinked Functional Bilayer Hydrogel Modulates Local Microenvironment and Remyelination After Optic Nerve Injury
Source: Adv Sci (Weinh). 2026 Sep 27:e78031. Online ahead of print. doi: 10.1002/advs.78031 (PMC13616303; doi:10.1002/advs.78031)
Supplement: Supplementary file 1 [file ADVS-9999-e78031-s001.docx]

Supporting Information

**In Situ Photo‑Crosslinked Functional Bilayer Hydrogel Modulates Local Microenvironment and Remyelination after Optic Nerve Injury**

Tonghe Pan^1,2,3,4,5†^, Yate Huang^3,4,5†^, Junhao Zhang^3,4^, Tian Shudong^3,4,5^, Zhixing Li^3,4^ , Xuanwen Li ^3,4,5^,Yanchen Zhang^3,4^, Dongmei Wang^1,2,3,4,5^,Sihao Ye^1,2,3,4,5^, Ming Jin^3,4,5^, Neng Ling^2,3,4,5^, Wentao Yan^3,4,5,*^, Kaihui Nan^3,4,*^, Wencan Wu^1,2,3,4,5,*^

^1^ Eye Research Center, Hangzhou Institute of Medicine, Chinese Academy of Sciences, Eye Hospital, Wenzhou Medical University, Hangzhou, Zhejiang 310018, China

^2^ Oujiang Laboratory (Zhejiang Lab for Regenerative Medicine, Vision and Brain Health), Wenzhou, Zhejiang 325000, China.

^3^ State Key Laboratory of Eye Health, Eye Hospital, Wenzhou Medical University, Wenzhou, Zhejiang 325027, China

^4^ National Engineering Research Center of Ophthalmology and Optometry, Institute of Biomedical Engineering, Eye Hospital, Wenzhou Medical University, Wenzhou, Zhejiang 325027, China

^5^ Zhejiang Key Laboratory of Key Technologies for Visual Pathway Reconstruction

^†^ These authors contributed equally to this work.* Corresponding author

E-mail: yanwentao0625@163.com (W. Y.); nankh@wmu.edu.cn (K. N.); wuwencan@wmu.edu.cn (W. W.)

Fig. S1. Swelling ratio of GelMA hydrogels over time in PBS at 37 ℃.

Fig. S2. *In vitro* degradation behavior of GelMA hydrogels in the presence of collagenase (1 μg/mL) under a mi-croinjection system at 37 ℃.

Fig. S3. N₂ adsorption–desorption isotherm and pore size distribution of MPDA.

Fig. S4. Live/dead cell staining of R28 Cells under different treatments (scale bars: 100 μm).

Fig. S5. Representative immunofluorescence images of P65 or IκBα in LPS-induced microglia under different treatments (scale bars: 50 μm).

Fig. S6. Representative Western blot bands and quantitative analysis of IL-1β, caspase-1, and NLRP3 in microglia under different treatments (n = 3). **p* < 0.05, ***p* < 0.01, ****p* < 0.001.

Fig. S7. Representative fluorescence images of CTB-labeled axons in the optic nerve post-PONI.

Fig. S8. Whole-retina fluorescence images following Brn3a immunofluorescence staining (yellow dashed lines demarcate the directly injured and secondary degeneration areas).

Fig. S9. Quantitative fluorescence intensity decay curve and fitted retention profile of GO/GI-Rhod at the optic nerve following local application (n = 3).

Fig. S10. Representative fluorescence images showing the progressive *in vivo* degradation of the locally applied hydrogel after optic nerve injury (The white polygon indicates the incompletely degraded bilayer hydrogel; scale bars: 200 μm).

Fig. S11. High-magnification TEM images and morphometric analysis of myelin sheaths in optic nerve cross-sections under different treatments. (A) Representative high-magnification TEM images. (B) Percentage of myelinated axons. (C) g-ratio.

Fig. S12. Toluidine blue staining of optic nerve cross-sections under different treatments.

Fig. S13. Schematic illustration of the pupillary light reflex test and quantitative analysis of pupil constriction at 8 weeks post-PONI under different treatments. **p* < 0.05, ***p* < 0.01, ****p* < 0.001.
